# Performance of Nanocomposite Membranes Containing 0D to 2D Nanofillers for CO_2_ Separation: A Review

**DOI:** 10.3390/membranes8020024

**Published:** 2018-05-14

**Authors:** Saravanan Janakiram, Mahdi Ahmadi, Zhongde Dai, Luca Ansaloni, Liyuan Deng

**Affiliations:** Department of Chemical Engineering, Norwegian University of Science and Technology (NTNU), NO-7491 Trondheim, Norway; saravanan.janakiram@ntnu.no (S.J.); mahdi.ahmadi@ntnu.no (M.A.); zhongde.dai@ntnu.no (Z.D.)

**Keywords:** hybrid membranes, nanocomposite membranes, CO_2_ capture, nanofillers, gas separation

## Abstract

Membrane technology has the potential to be an eco-friendly and energy-saving solution for the separation of CO_2_ from different gaseous streams due to the lower cost and the superior manufacturing features. However, the performances of membranes made of conventional polymers are limited by the trade-off between the permeability and selectivity. Improving the membrane performance through the addition of nanofillers within the polymer matrix offers a promising strategy to achieve superior separation performance. This review aims at providing a complete overview of the recent advances in nanocomposite membranes for enhanced CO_2_ separation. Nanofillers of various dimensions and properties are categorized and effects of nature and morphology of the 0D to 2D nanofillers in the corresponding nanocomposite membranes of different polymeric matrixes are discussed with regard to the CO_2_ permeation properties. Moreover, a comprehensive summary of the performance data of various nanocomposite membranes is presented. Finally, the advantages and challenges of various nanocomposite membranes are discussed and the future research and development opportunities are proposed.

## 1. Introduction

The continuous increase of CO_2_ concentration in the atmosphere engenders an urgent call for reducing CO_2_ emissions, with the highest contribution coming from the industrial sector [1]. Although conversion of industrial processes to more environmental friendly options is a viable long-term solution, a fast implementation of CCS (Carbon Capture and Storage) is considered the most effective way to reduce emissions in the short-term. However, the development of more efficient CO_2_ capture technologies is crucial for CCS deployment in order to reduce the capture costs and meet economic feasibility standards [2]. Polymeric membranes are a promising alternative to traditional capture technologies (e.g., amine-based absorption and solid adsorption), in view of advantages such as absence of dangerous emissions or need of harmful chemicals, lower footprint, reduced energy requirement and modularity. Membranes can be applied in syngas (CO_2_/H_2_) purification, gaseous biofuels upgrading (CO_2_/CH_4_ and CO_2_/H_2_), and CO_2_ capture from post combustion flue gas (CO_2_/N_2_). However, high separation performance are needed to make the technology competitive [3] at industrial scale, thereby driving a continuous material development.

Typically, the separation properties of a gas separation membrane are characterized in terms of gas permeability and selectivity. Gas permeability (*P_A_*) is defined as the flux per unit area (*J_A_*) of a permeating component scaled on its driving force across the membrane (typically identified as partial pressure difference (Δ*p_A_*) between upstream and downstream side) and on the membrane thickness (*ℓ*):

(1) $$P_{A}=\frac{J_{A}\cdot \ell }{\Delta p_{A}}$$

Permeability is frequently reported in Barrer (1 Barrer = 10^−10^ cm^3^ (STP) cm^−1^ s^−1^ cmHg^−1^ = 3.346 × 10^−16^ mol m^−1^ Pa^−1^ s^−1^).

Solution-diffusion is the most common mechanism used to describe the gas transport through a membrane: gas molecules are initially absorbed on the upstream side of the membrane, diffuses across the dense layer and then desorbed on the downstream side [4]. On the other hand, facilitated transport occurs in presence of moieties that reversibly react/complex with a target gas penetrant, and the chemical reaction/complexation contributes to its transport [5].

Lately, many efforts have been dedicated to the fabrication of defect-free sub-µm thick membranes, in order to maximize the flux for a given membrane material. In this case, the selective layer is coated on a porous support to ensure the mechanical resistance, and the transmembrane flux is frequently described referring to Permeance, which is defined simply as the flux scaled on the driving force. The permeance is often reported in GPU (gas permeation unit, 1 GPU = 10^−6^ cm^3^ (STP) cm^−2^ s^−1^ cmHg^−1^ = 3.346 × 10^−10^ mol m^−2^ Pa^−1^ s^−1^). Selectivity (*α*) for gaseous mixture is defined as:

(2) $$\alpha =\frac{y_{A}/y_{B}}{x_{A}/x_{B}}$$

where *y* and *x* are the molar fractions of the gases *A* and *B* in the permeate and the feed, respectively. Equation (2) can be approximated to the ratio of the permeability values if the downstream partial pressure of each component can be considered negligible compared to the upstream value [6].

**Figure 1:**
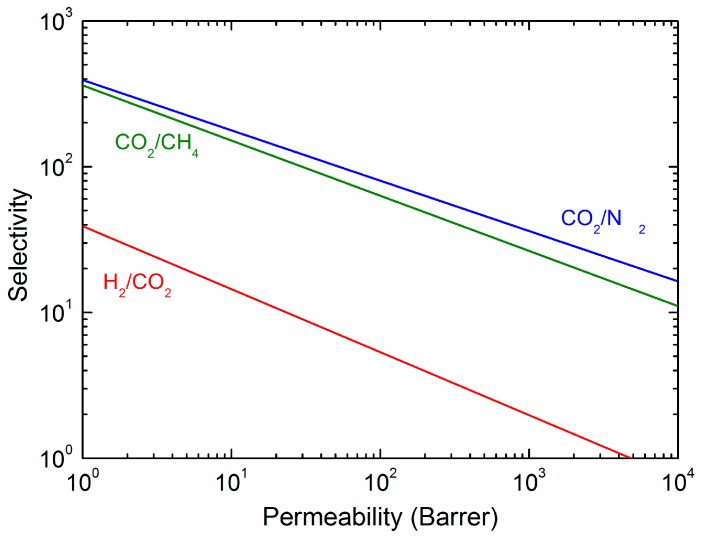
Robeson upper bounds for the three gas-pairs of interest for CO_2_ capture applications (CO_2_/N_2_, CO_2_/CH_4_ and H_2_/CO_2_). The permeability on the x-axis refers to the most permeable gas component (CO_2_ for CO_2_/N_2_ and CO_2_/CH_4_; H_2_ for H_2_/CO_2_).

Polymeric materials, where permeation takes place according to the solution-diffusion mechanism, are characterized by a trade-off between permeability and selectivity, which is well described by the Robeson upper bound [7]. As shown in Figure 1, an increase in gas permeability of a membrane corresponds typically to a decrease in its selectivity and vice versa. The upper bound represents also the state-of-the-art membranes for separation of a given gas pair. In the past 10 years, overcoming the upper bounds of polymeric membranes has become the main goal of many membrane scientists. The following strategies are typically adopted to overcome the trade-off between permeability and selectivity: (i) development of materials with high free volume and high selective features (e.g., thermally rearranged-polymer and polymers of intrinsic microporosity (PIMs) [8]); (ii) introduction of reactive carriers to provide facilitated transport; (iii) addition of fillers to improve the gas transport by enhancing the solubility or diffusivity of gases in membranes. The latter strategy, also known as “hybrid membranes”, has been demonstrated to be a successful approach, as it allows to exploit the transport properties of phases with different nature [9,10].

Micro- or nano-size particles have been largely used as fillers to fabricate hybrid membranes, as they typically own a higher permeability and/or selectivity compared to polymeric materials, or their surface properties can enhance the transport of target gases. At the same time, their dispersion in the polymer matrix allows an easier exploitation of the properties of these phases, as purely inorganic matrix is typically very brittle and difficult to manufacture. Hybrid membranes have been thoroughly reviewed from various aspects, including two very recent studies with focus on CO_2_ capture and mass transfer structure [9,10]. Recently, liquid phases (usually with negligible vapor pressure) have also been applied to prepare hybrid membranes with polymeric materials in order to enhance the performance [11].

Based on the contribution of the fillers to the gas transport, it is possible to categorize hybrid membranes as mixed matrices and nanocomposites [6]. In nanocomposite membranes, the fillers are typically used to enhance the solubility selectivity by the addition of nano-sized fillers with high affinity towards the penetrants (surface adsorption) or the presence of surface moieties on the fillers that can reversely react with the gases. Moreover, nanofillers steer the spatial distribution of polymeric chains and hence their orientation in the bulk matrix, possibly leading to the increase in free volume (formation of voids at the interface [6]). In many cases, nanofillers also increase the mechanical and chemical stability of the polymeric membrane. On the other side, phases embedded in mixed matrix membranes, in general, exhibit superior solubility and/or diffusivity compared to the bulk polymer matrix. The fillers used in these membranes are usually porous, and the pore structure and architecture of the fillers are typically tailored prior to their incorporation into the polymeric matrix. In both cases, the mass transport through the hybrid matrix is not only merely driven by solution-diffusion, but also other mechanisms like Knudsen diffusion, surface diffusion or molecular sieving, depending on the filler’s nature.

**Figure 2:**
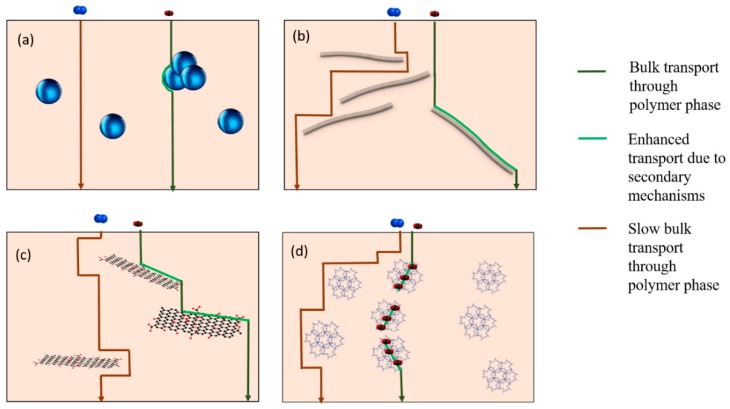
Representative transport pathways in hybrid membranes with (**a**) 0D (**b**) 1D (**c**) 2D (**d**) 3D fillers.

Table 1 lists the types and classification of nanofillers used for fabricating hybrid membranes. The typical gas transport pathways in hybrid membranes with different nanofillers are represented in Figure 2. In the light of emphasizing the role of nanofillers in nanocomposite membranes according to their morphology and transport mechanisms, they are classified into zero-dimensional (0D), one-dimensional (1D) and two-dimensional (2D) fillers. When the secondary transport mechanisms through the embedded phase considerably contribute to the overall flux of the penetrant in the hybrid matrix, the nanoparticles are classified as three-dimensional (3D) fillers, and the membranes are labelled under the class of mixed matrices. 

The present review provides a comprehensive overview of the latest studies on nanocomposite membranes for CO_2_ separation and a discussion of the mechanism behind the performance improvement, aiming at finding effective strategies for the development of highly efficient CO_2_ separation membranes. This review will only focus on nanocomposite membranes fabricated using 0D to 2D nanofillers. For each filler, the membrane performances are analyzed in view of the expected influence on the transport properties. Different polymeric phases are also compared to identify the most promising hybrid compositions. CO_2_ permeability and selectivity (CO_2_/N_2_ and CO_2_/CH_4_) have been tabulated as a function of filler loading to facilitate easier performance evaluation of the hybrid membranes. If numerical values were not reported in the original article, the graphs were digitalized (WebPlotDigitizer, Version 4.1) to extract relevant information. Finally, an outlook on future research directions is provided based on the advantages and challenges of the investigated nanocomposite membranes.

## 2. Zero-Dimensional (0D) Fillers

### 2.1. Si-Based Materials

The addition of nonporous fillers to polymers was initially perceived to decrease the permeability and have little effect over the selectivity [12,13]. This was attributed to additional hindrance in penetrant diffusion pathways that emerge by incorporating nonporous inorganic fillers. Conflictingly, the study by Moaddeb and Koros [14] established simultaneous increase in oxygen/nitrogen selectivity and oxygen permeability when silica particles were added to dense 6FDA-IPDA films. The adsorption of the polymer chains to the surface of silica particles increased the rigidity of the polymer matrix, leading to enhanced selectivity by increasing the energetics of diffusion. On the other hand, the presence of SiO_2_ particles induced disruption of polymer chain packing. After their pioneering work, many efforts have been dedicated to the addition of silica-based materials to polymeric membranes for different gas separation applications.

In the last years, several silica-based nanofillers have been studied for hybrid membranes in CO_2_ capture [15,16,17,18,19]. The most notable ones are silica nanoparticles, fumed silica and POSS. Both SiO_2_ nanoparticles and fumed silica have been found to increase solubility of gases in the hybrid matrix while negatively affecting their diffusivity [15]. Table 2 summarizes some representative hybrid materials of silica-based nanofillers. Cong et al. summarized a detailed study of silica-based nanocomposites with special focus on polyimides [20]. Here in, some representative silica-based hybrid membranes for CO_2_ capture were reported. Shen and Lua [15] studied the effect of functionalization prior to incorporation of silica nanoparticles in P84 (co-polyimide BTDA-TDI/MDI) matrix. Silica nanofillers functionalized with APTES were dispersed in solution of P84 by means of ultrasonication. The addition of nanoparticles increased the density of the membrane and decreased the fractional free volume when compared to unmodified silica hybrids due to strong interaction of the hydrophobic groups with the polymer chains. However, at a higher loading of about 25 wt %, the modified silica particles aggregated, forming large non-selective continuous voids around the aggregates. This led to an increase in CO_2_ permeability by three folds while compromising the selectivity over five times compared to that of the neat polymer membrane (Table 2). Yu et al. [21] homogeneously dispersed silica nanoparticles in PEBAX^®^ 1657 (crosslinked polyether block amide) casting solution by mere stirring at high speed. The gas permeation results of the prepared membranes showed the loss of CO_2_ solubility with increasing silica nanoparticles loading in polymer matrix due to the decrease in polymer free volume. Although the diffusivity increased slightly when increasing nanofiller loading, the CO_2_ permeability of the membrane dropped with simultaneous decrease in CO_2_/N_2_ ideal gas selectivity. At about 30 wt % loading, the CO_2_ permeability decreased from 80 Barrer to 52 Barrer with a reduction in CO_2_/N_2_ gas selectivity from 70 to 46. Sadeghi et al. [22] prepared silica hybrid composites by mixing polyurethane with silica sol synthesized by hydrolysis from tetraethoxysilane. They revealed that the silica nanoparticles preferentially distributed in soft segments of poly urethane polymer matrix. However, it was initially assumed from the entropic point of view that the silica particles would prevent formation of crystals in the hard segments. This led to a drop in CO_2_ permeability from 190 Barrer of the neat membrane to 125 Barrer at a loading of 20 wt %, while CO_2_/N_2_ gas selectivity increased from 25 to 40 and CO_2_/CH_4_ gas selectivity from 10 to 13, respectively. Additionally, the permeation in hybrid membranes was modeled by Higuchi model, which exhibited good correlation with the experimental results [22]. Similar results were obtained when the nanoparticles were loaded in a blend of polycaprolactone-based polyurethane membranes [16], where the reduction in permeability was mainly due to the decrease in free volume and chain mobility of the soft segments and the generation of tortuous diffusive pathways for penetrants. Azizi et al. [17] dispersed silica in PEBAX solution by sonication. They found that increase in CO_2_ solubility upon the addition of SiO_2_ nanoparticles increased the CO_2_ permeability from 110.7 in neat PEBAX-1074 (crosslinked polyether block amide) matrix to 152.1 Barrer with the nanoparticle loading of 8 wt % in the membrane, with slight improvement in CO_2_/N_2_ selectivity from 11.1 to 13.3. In another recent study, Kim and co-workers [19] developed novel thin composite hybrid membranes with functionalized silica particles in PEG-based selective layer. The silica nanoparticles coated with PDA and PEI increased the voids generated at the interface, thereby enhanced the CO_2_ permeation; CO_2_ permeance of up to 2150 GPU and CO_2_/N_2_ selectivity of 39 were obtained at 5 wt % loading of the functionalized silica nanoparticles. 

Chen et al. [23] successfully fabricated hybrid Matrimid^®^ (BTDA-DAPI polyimide) membranes containing silica nansopheres synthesized through sol-gel mechanism using different precursors. With the addition of 3.11 wt % of silica gel obtained from tetraethoxysilane, the CO_2_ permeability increased almost 5 folds (from 9.9 Barrer to 46.3 Barrer) compared to the neat polymer, with a loss in CO_2_/CH_4_ selectivity from 35.3 to 28.3. Similar results were obtained in the case of silica particles synthesized from tetramethoxysilane and tetrapropoxysilane precursors although they were treated at different temperatures. The addition of silica nanoparticles was also found to increase the rigidity of polymer matrix and waive the plasticization effect.

Polyhedral oligomeric silsesquioxanes (POSS) represent a promising class of silica-based nanoparticles. POSS are compounds of general formula [RSiO_3/2_]_n_, where n = 6–12, and R is either hydrogen or an alkyl, olefin, alcohol, acid, amine, epoxy or sulfonate group. These compounds constitute a cage-like structure, which is often cubic, or with a hexagonal, octagonal, decagonal, or dodecagonal prism-shape [24]. Although POSS has a caged structure extending in three-dimensions, in this review, POSS is classified under 0D nanofillers as no studies exist proving secondary mechanisms of CO_2_ transport through the caged structures to the best of our knowledge. POSS compounds gained much wider attention owing to the tunable dispersibility and compatibility with polymeric solutions through a variety of functional groups that can be chemically bonded to the POSS structure. These reactive functional groups can be used to create physical/chemical linkages with the polymeric chains of the bulk matrix. Several researches claimed CO_2_ transport through POSS particles by molecular sieving in hybrid matrices due to the presence of caged structure. However, this is not substantiated with studies so far [24]. Chua et al. [25] cross-linked polyetheramine (PEA) with epoxy-POSS that resulted in a hybrid matrix obtained via sonication. The cross-linked structure helped in decreasing the crystallinity of overall matrix by disrupting PEO segment packing with increasing POSS content. The reduced crystallinity of PEO chains due to crosslinking by inorganic POSS also helped in increase CO_2_ permeability to over 335 – 380 Barrer at a pressure of 1 bar with 50 wt % POSS loading, while the neat polymer matrix had a CO_2_ permeability of only 13 Barrer when measured at 35 °C and 4.5 bar upstream pressure. The CO_2_/N_2_ ideal gas selectivity of the hybrid membrane simultaneously increased to up to 50 in the same pressure range [25].

Functionalization of POSS with PEG has been reported [26] and the nanoparticles have been embedded in two types of PEBAX (crosslinked polyether block amide) matrices through physical blending. The study revealed interesting effects of the matrix filler interactions on gas permeation properties. Distribution of nanofillers was found to be more homogeneous in case of PEBAX^®^ 1657 than in PEBAX^®^ 2533 due to the incompatibility of PEG-POSS with Polyimide-12 and PEO components in the latter matrix. This led to the increasing surface roughness in PEBAX^®^ 1657, whereas the opposite trend is observed in the case of PEBAX^®^ 2533. The increase of gas solubility in the hybrid matrix caused by the higher surface roughness and the presence of CO_2_-philic moieties (i.e., PEG) resulted in an enhancement of the CO_2_ permeability of PEBAX^®^ 1657 from about 70 Barrer to 150 Barrer at 30 wt % filler loading with negligible change in selectivity. PEG-functionalized POSS were also added to high free volume polymer PIM-1 by Yang et al. [27]. The nanoparticles were dispersed in the polymer cast solution in chloroform via stirring followed by sonication to obtain a homogeneous dispersion. The high compatibility of these fillers with PIM-1 matrix led to the blockage of the ultra micropores of PIM-1 and the rigidification of the polymer chains. Consequently, decrease in diffusivity was reported with increasing filler loading with a slight increase in CO_2_ solubility. Addition of 10 wt % PEG POSS nanoparticles decreased the permeability from 3795 Barrer to 1309 Barrer while increasing the CO_2_/N_2_ and CO_2_/CH_4_ selectivity from 19 and 12 to 31 and 30, respectively. Furthermore, over a period of 30 days, ageing phenomena were significantly reduced due to the addition of 10 wt % nanoparticles within the polymer matrix. This effect has been related to the rigidification of polymer chains surrounding the filler interface.

Recently, an interesting study by Kinoshita et al. [28] further underscored the effect of the functionalization of POSS when added to PIM-1. The authors prepared hybrid membranes with unmodified POSS, amine-functionalized POSS and nitro-functionalized POSS. The functionalization helped in improving the dispersion of POSS in polymer matrix after sonication as the unmodified POSS merely increased the permeability with the loss of selectivity due to incompatibility. Amine-functionalized POSS led to a selectivity enhancement by about 200% for CO_2_/N_2_ and 250% for CO_2_/CH_4_ at 20% POSS loading in a nanocomposite membrane, with a slight decrease in CO_2_ permeability. The enhancement of selectivity is attributed to the large affinity of amine and cyano groups in PIM-1, leading to increased hydrogen bonding and thus increased surrounding polymer chain rigidity. On the other hand, nitro-functionalized POSS slightly increased the selectivity at the expense of minor CO_2_ permeability losses with increasing loading. It was found that the greater interaction of POSS fillers with the polymer matrix led to the retention of fractional free volume for a longer period. Therefore, the amine and nitro-functionalized POSS significantly slowed down the ageing phenomena in PIM-1 with CO_2_ permeability dropping by only 35% after 90 days. Functionalization of POSS with amino- and amidino-moieties has also been recently reported for the fabrication of facilitated transport membranes [29]. However, despite the successful embedding of the nanoparticles in the hydrophilic polyvinyl alcohol (PVA) matrix, the membrane performance did not improve under fully saturated conditions.

### 2.2. Metal Oxides Materials

Application of metal oxide particles as reinforcements or nanofillers in nanocomposite materials requires the controlled synthesis of the particles to achieve the targeted properties. Most of the metal oxide particles are prepared using the sol-gel technique, which exploits nucleation, growth and aging mechanisms to tune the properties of the resulting nanoparticles. Monodisperse particles systems with narrow particle size distribution is usually desired for applications as nanofillers. Some applications have also documented the use of micro aggregates of metal oxide particles as nanofillers [30,31,32]. The control of aggregation in nanoparticles during the synthesis stage has been well studied in the field of surface and colloids [33,34]. However, the dispersion of particles in polymeric solutions that are less than 100 nm in size is still a challenge as the surface interactions become much stronger [35]. The challenge is even more problematic when the concentration of nanoparticles in the bulk matrix is increased. Most metal oxides exhibit intrinsic affinity towards polar gases like CO_2_, leading to increased penetrant solubility in the hybrid matrix. When compared to silica nanoparticles, metal oxides display a lower tendency for agglomeration [32]. Hence, with homogenous distribution of the embedded phase, chances of particles aggregation and defects formation during the process of membrane fabrication are expected to be significantly reduced. Furthermore, the surface properties in metal oxides (e.g., TiO_2_) open up possibilities of functionalization to further enhance the filler-specific properties in the hybrid matrix.

#### 2.2.1. TiO_2_-Based Nanocomposite Membranes

**Figure 3:**
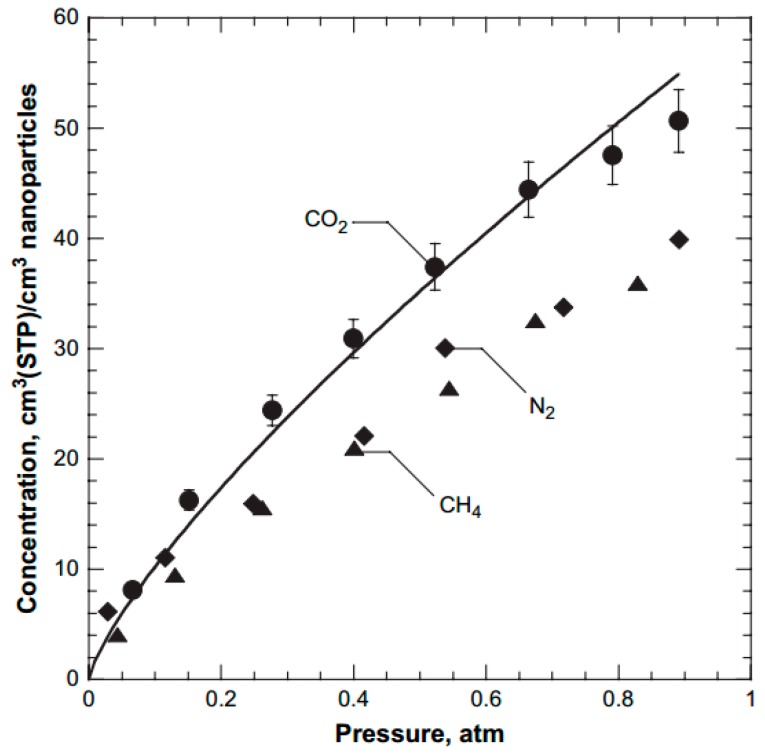
Gas uptake capacity of TiO_2_ at 35 °C. Solid line indicates corresponding Freundlich isotherm [30].

The use of TiO_2_ in nanocomposite membranes for gas separation is traced back to the pioneering work of Hu et al. [36]. They found that the activation energy of permeation for specific gases like CO_2_ and H_2_ is lowered in the hybrid membrane due to the interactions with TiO_2_ present in the polymeric phase. Their intrinsic affinity towards CO_2_ results in adsorption capacity that is several folds higher compared to the uptake capacity of conventional polymers. For instance, Brookite (TiO_2_) nanoparticles were found to adsorb 20 times more than an equivalent volume of pristine PTMSP polymer at 35 °C and 1 bar [32]. Figure 3 shows the pure gas adsorption isotherms of various gases in TiO_2_. Owing to their low cost and high CO_2_ adsorption capacity, TiO_2_ nanoparticles were used as nanofillers for nanocomposites involved in CO_2_ separation.

Nanoscale aggregates of TiO_2_ (primary particles as small as 3 nm) were added to increase the separation performance of polymeric membranes. Most researchers documented increase in CO_2_ permeability due to the increased diffusion and/or solubility coefficients (mostly at high loadings). For high free volume polymers like poly(trimethylsilylpropyne) PTMSP, poly(trimethylgermylpropyne) (PTMGP) and polymethylpentene (PMP), the diffusion coefficients of different gases were found to increase at higher TiO_2_ loading in the matrix. Thus, the permeability of pure gases also followed a similar trend. For instance, addition of nanofillers increased CO_2_ permeability from 35,000 to 71,000 Barrer in case of PTMSP with no drastic decrease of selectivity (as shown in Table 3) [37].

**Figure 4:**
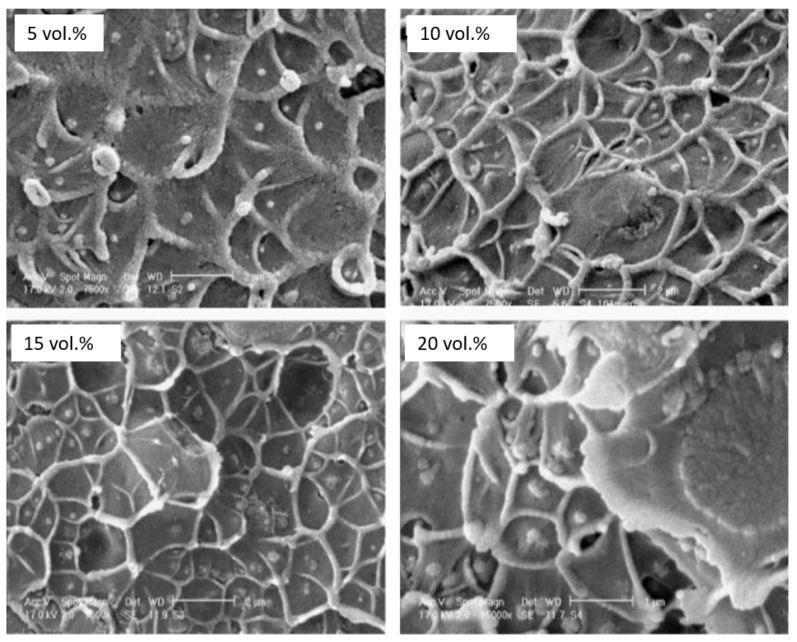
SEM micrographs of TiO_2_ hybrids of Matrimid^®^ indicating presence of interfacial voids [41].

Yave et al. [38] developed novel methods to fabricate PTMGP membranes with varying cis/trans ratio in the polymer chain and studied the effect of adding TiO_2_ on the permeation and stability of the membranes. TiO_2_ particles of around 10 nm diameter were added as a sol to polymer solution through constant stirring followed by solvent evaporation to obtain nanocomposite membranes. The addition of TiO_2_ was found to marginally increase the permeability of CO_2_ by approximately 30% with respect to the neat polymer by addition of 10 wt % of the filler, with positive impact on the gas selectivity (Table 3). The ageing phenomenon slowed down with the addition of nanofillers. At a higher loading (20 wt %), however, the drop in performance was observed. Matteucci and his co-workers [32] devised a much detailed study on the effect of TiO_2_ on physical properties of PTMSP hybrids and their performance. The authors dispersed TiO_2_ nanoparticles by mixing and characterized the polymer-particle interaction with AFM and TEM, highlighting the details of the polymer/particle interface. The density of the hybrid matrix was remarkably lower than the predicted additive density due to the generation of interface voids between the filler and the polymer matrix, especially at a higher loading (about 33 vol %). As shown in Table 3, this effect has led to an increase in the CO_2_ permeability (up to 71,000 Barrer) with limited effect on the membrane selectivity. Shao et al. [39] studied the performance of hybrid cross-linked PMP containing TiO_2_ particles, which were used in order to compensate the losses in permeability upon crosslinking. Nanoparticles were sonicated in CCl_4_ and then the polymer with the cross linker was dissolved in the sol to obtain a stable dispersion for fabricating membranes by solvent evaporation. It was found that the observed permeability enhancement was related to the increased free volume associated to the disruption of polymer chain packing. Additionally, the size of the nanofillers influenced the change in permeability. Smaller particles engender bigger positive effect due to the larger polymer-particle interfacial area. Nevertheless, the study presented outstanding long-term stability in terms of permeation properties with flux almost constant for about 175 days. The effect of TiO_2_ nanoparticles was investigated also for other glassy polymers, such as poly (ether sulfone) (PES) [40] and Matrimid^®^ (BTDA-DAPI polyimide) [41]. Although stable dispersions of particles in polymeric solutions were obtained with different loading through stirring/sonication, SEM micrographs of the resulting membranes revealed the existence of large unselective interfacial voids upon drying (as shown in Figure 4). This led to an increase in gas permeability (due to larger diffusion coefficients) and the detriment of the selective features of the membrane.

In another study, Matteucci et al. [30] established the contrasting effect of TiO_2_ particles on gas diffusivity in rubbery polymer matrix—1,2-polybutadiene. The nanocomposite membranes were obtained by stirring nanoparticles in polymer solution followed by solution casting. The presence of TiO_2_ nanofillers still contributed to increasing voids as confirmed with the density changes. However, the diffusivity of gases was found to decrease when nanofillers were added and was lower than pristine polymer even at 27 vol % loading. Although the effects on the diffusivity was contrasting when compared to glassy polymers, a 210% increase in the CO_2_ permeability of the nanocomposite was observed at the same loading (Table 3). This effect is attributed to the increased gas solubility, leading to virtually no change in selectivity with the addition of nanofillers. Azizi et al. [42] reported the TiO_2_ hybrid with blend matrix of PEBAX-1074 (crosslinked polyether block amide) and PEG 400. The PEG400 is used as plasticizer in order to reduce the crystallinity of the PEBAX matrix. Increasing TiO_2_ content led to the disruption of hydrogen bonding in the PA segment, resulting in reduced crystallinity in the hybrid matrix as confirmed by X-Ray Diffraction (XRD) technique. This reduced crystallinity fostered increase in CO_2_ permeability from 150 Barrer to 205 Barrer and CO_2_/CH_4_ selectivity from 21 to 24 simultaneously. The authors obtained similar results with neat PEBAX-1074 matrix without PEG [17].

Interestingly, the addition of TiO_2_ nanoparticles has also been reported to have a negative effect on the gas permeability of polyvinyl alcohol (PVA)-based hybrid matrix: even at a nanofiller loading of 40 wt %, the permeability of CO_2_ did not surpass the performance achieve by the neat polymer and the effect on the gas selectivity was slim [43]. Nevertheless, increasing the TiO_2_ content in the polymer matrix increased the tensile strength and thermal stability of the membrane. Contrastingly, when the same nanofillers were loaded in polyvinyl acetate (PVAc), the authors reported positive effect on the gas transport properties, with an increase from 2.9 to 5.8 Barrer. The reduction in primary crystallinity of PVAc matrix led to increased permeability through faster diffusion. In addition, the glass transition temperature increased with the loading of nanofillers [44].

The use of TiO_2_ in water-swelling polymers and the functionalization of TiO_2_ nanofillers prior to incorporation in polymer matrix were first demonstrated by Xin et al. [31] with sulfonated poly(ether ether ketone) (sPEEK). Stable dispersions of both functionalized and non-functionalized TiO_2_ fillers in polymer solution were obtained by stirring. The free volume of the hybrid matrices increased marginally due to the creation of voids upon the addition of nanofillers. This free volume variation led to enhanced water uptake from about 11% in the neat polymer to 25–28% in the matrix containing 15 wt % of TiO_2_ nanospheres. At this high loading of unmodified TiO_2_ nanofillers, the humid gas permeability almost doubled due to the increase in both diffusion and solution, as the selectivity slightly decreased. Furthermore, the functionalization of the TiO_2_ nanofillers with amine groups (Dopamine and Dopamine-PEI) enhanced the permeability and selectivity, which is believed to be due to the introduced functional groups contributing to CO_2_ facilitated transport. The regain of selectivity that dropped with un-functionalized TiO_2_ is due to the lower affinity of N_2_ and CH_4_ to amine-containing nanofillers

#### 2.2.2. Other Metal Oxides

Zinc oxide is a commonly used nanofiller for nanocomposite membranes, especially in ultrafiltration and desalination membranes, owing to its photo-catalytic and anti-bacterial properties [45]. The use of Zinc oxide particles in polymeric membranes for CO_2_ separation is ascribed to their intrinsic affinity towards CO_2_ and high adsorption potential [46,47]. As a base element for several zeolites and MOFs, Zinc is nowadays used in various mixed matrix membranes [37,48]. However, ZnO as impermeable nanofiller for gas separation membranes is seldom studied. It was reported that the crystallinity of PEBAX (crosslinked polyether block amide) matrix was significantly reduced in presence of ZnO nanoparticles due to the disruption of inter-chain hydrogen bonding between the polyamide segments [45]. Similar results were obtained by Azizi et al. [49] who dispersed ZnO particles in Dimethylformamide. The study also reported that modification of nanofiller surface by Oleic acid enhanced the stability of the dispersion. With the addition of modified fillers, the mechanical and thermal stability of the membranes were found to increase, as is in general the case of inorganic fillers. The permeability was also found to increase from 110 to 152 Barrer (Table 3) with increasing ZnO loading in PEBAX^®^-1074 membranes, with a slight increase in selectivity [49]. However, in PEBAX-PEG system the selectivity showed a moderate reduction with increase in permeability from 72 to 95 Barrer, which might be due to the minimal influence of ZnO on PEG chain packing. The different affinity of ZnO towards CO_2_ from that towards N_2_ and CH_4_ contributes to further enhanced selectivity than that by adding most of other metal oxides, and the effects increases with increasing ZnO loading. A comparison of the effect of different metal oxide nanoparticles (SiO_2_, TiO_2_ and Al_2_O_3_) [17] embedded in PEBAX-1074 revealed that the greatest improvement both in terms of permeability and selectivity can be achieved by dispersing Al_2_O_3_ in the membrane matrix. The higher adsorption capacity of Al_2_O_3_ compared to other fillers resulted in a larger CO_2_ solubility at high loading of nanofiller and the formation of interface voids led to an increase of the diffusion coefficient. 

Magnesium oxide (MgO) is another interesting metal-oxide nanofiller for nanocomposite membrane fabrication. When compared to other metal oxide fillers, MgO has been reported to possess higher tendency of agglomeration in polymeric dispersions. Hence, in most cases, sonication of nanofillers in higher polymer concentration was used to obtain stable casting solutions. As shown in Table 3, for all the three pristine glassy polymers (Polysulfone (PSU), Matrimid^®^ (BTDA-DAPI polyimide) and PTMSP), the addition of MgO is found to increase the permeability along with the loading, but typically the variation took place to the detriment of the selectivity feature of the membrane. In the case of PTMSP, an increase of 1 order of magnitude of the CO_2_ permeability was reported. The increase in free volume generated by the poor compatibility at the MgO-polymer interface was identified as the cause of the observed trends [50,51]. When compared to other metal-oxide inorganic fillers, MgO exhibits relatively poor performance in enhancing the membrane selectivity, probably due to a lower affinity with the investigated polymer phases, which generated larger interfacial voids. However, when embedded in high free volume polymer (PTMSP), the addition of MgO is found to have positive effect on aging with almost no change in permeability after a sufficient period of time [52].

**Figure 5:**
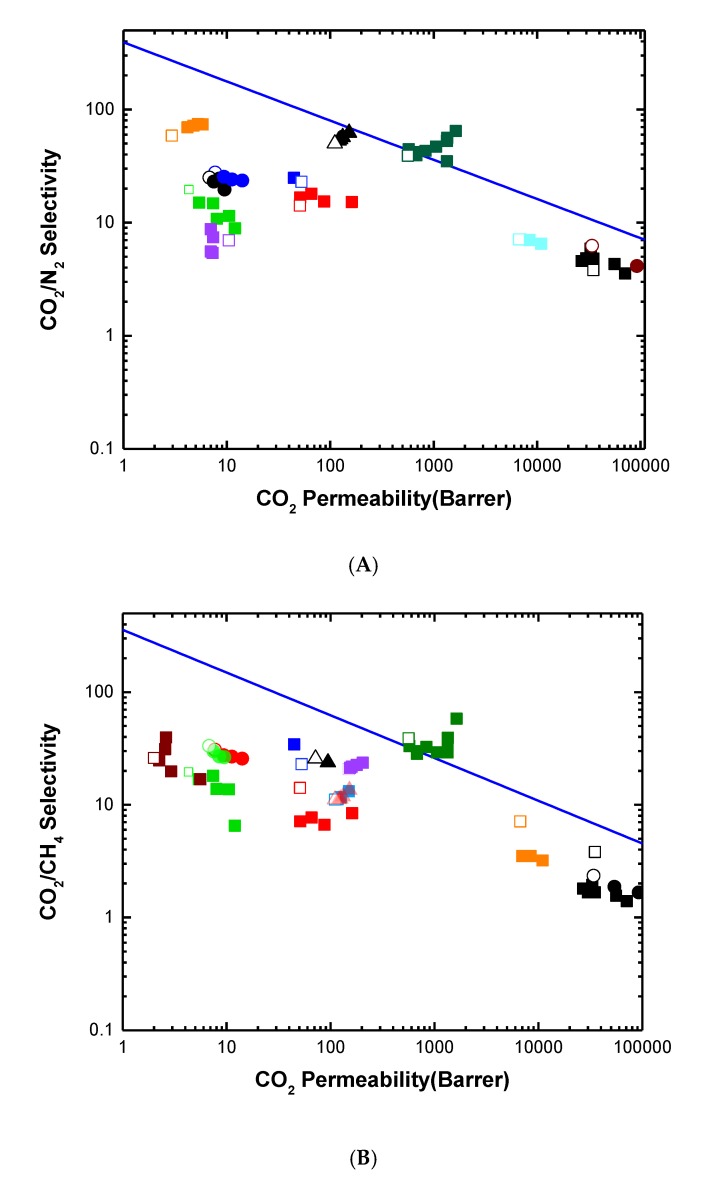
Robeson plot for Metal oxide-based hybrid membranes as a function of loading (A) CO2/N2 gas pair (B) CO2/CH4 gas pair. Squares (■) refers to TiO2-based membranes; circles (●) refers to MgO-based membranes; triangles (▲) refers to ZnO-based membranes. Empty shapes indicate neat polymer matrix and filled ones represent hybrid membranes. Different colors represent individual studies in each class of nanofillers (reported in Table 3).

The effect of adding metal oxide nanofillers in polymer matrices on the permeation performance is reflected in Robeson plots, as shown in Figure 5. In almost all cases, the increase in nanofiller loadings lead to an increase in permeability, particularly at high loadings, due to an increase of CO_2_ affinity with the hybrid matrix (increasing the CO_2_ solubility) or the generation of interfacial voids between the polymer and the embedded phase (improving the diffusivity). This was also accompanied by the increase in selectivity for TiO_2_-based membranes, thus moving towards an overall improvement of the separation performance of the hybrid membranes. In the case of ZnO and MgO nanofillers, in most cases, the formation of large non-selective voids at filler-polymer interface results in a selectivity drop. However, unlike TiO_2_, these metal oxides have not been widely investigated and the use of diverse polymer phases with respect to the one reported in this review may possibly lead to better compatibility between the polymer matrix and the nanoparticles, thus to better performance of the hybrid membranes.

## 3. One-Dimensional (1D) Fillers

One-Dimensional fillers are renowned in nanocomposites for enhancing structural stability of the polymeric matrices due to their high aspect ratio. Typically, 1D fillers are characterized by an extremely high aspect ratio between the length and the other two dimensions. In polymeric membranes, they also contribute to enhance the disruption of the polymeric chain packing along the lateral dimension, affecting the free volume of the polymer matrix. Their inherent CO_2_ sorption and transport abilities also contribute to increased interest in CO_2_ separation membranes. This section summarizes different types of 1D fillers used in nanocomposite membranes for CO_2_ separation.

### 3.1. Carbon Nanotubes (CNTs)

**Figure 6:**
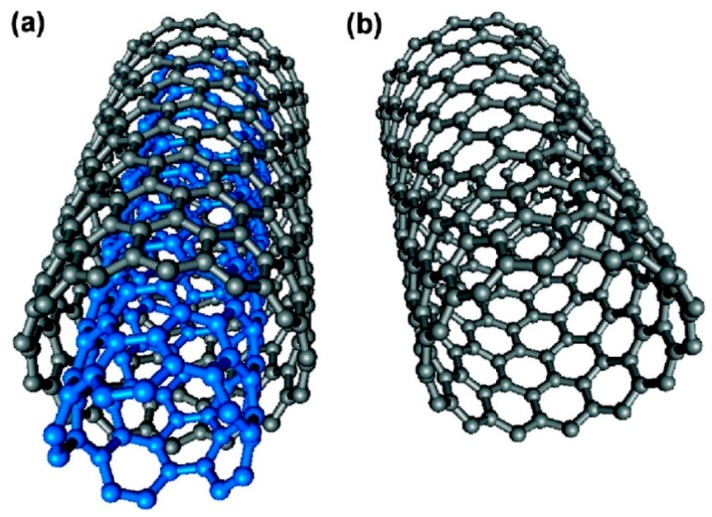
Multi-walled (**a**) and single-walled (**b**) CNTs (Adapted from [64]).

There is an increasing interest in the application of CNTs in sensors [53], composites [54], catalysis [55] and membranes for different types of separation [56,57]. CNTs are well known nanomaterials for their 1D feature as well as outstanding mechanical and thermal properties. Despite the low density (1.3–1.4 g/cm^3^), CNTs own an ultra-high strength-to-weight ratio which is in the order of 48,462 Nm/kg, surpassed only by the recently discovered colossal carbon tube [58,59]. The superior mechanical properties are derived from the sp^2^ carbon-carbon bond in the graphite layers of the structure, as seen in Figure 6, which leads to high stiffness and axial strength [60]. CNTs can present a high aspect ratio with inner core diameter as low as 4 Å and length up to micrometric size [61]. The synthesis of both single-walled carbon nanotubes (SWCNTs) and Multi-walled carbon nanotubes (MWCNTs) involves high temperature processes like chemical vapor deposition (CVD), carbon arc discharge, pulsed laser vaporization or high-pressure CO processes. This is followed by purification to separate non-nanotube impurities from the targeted nanotubes [62]. Recent methods for synthesis of ultra-long (as high as 18.5 cm) nanotubes have been documented [63]. SWCNTs possess high surface area with interstitial channels that give rise to various possible adsorption sites with high binding energy. On the other hand, MWCNTs contain interlayer spaces that might act as adsorption sites for smaller molecules. 

The use of CNTs in gas separation membrane was delved into after the theoretical study by Skoulidas et al. [65]. Their investigation reported transport diffusivities of light gases in CNTs to be exceptionally high when compared to zeolites of similar pore sizes. ZSM-12 and Zeolite Silicalite with a pore size of about 0.8 nm were used to compare the gas transport properties of CNTs with pore size of 0.81 and 1.36 nm, showing that the diffusion of light gases in SWNTs were much faster than in any microporous adsorbents, resulting in diffusivity ranges similar to gas diffusivities in liquids. The inherent smoothness of the nanotubes enables the high transport rate of light gases with diffusivities as high as in gases. This is attributed to the elastic collisions between the gas molecules and the wall due to its smoothness and the momentum along the wall remains undisturbed. Hence, the diffusion arises due to collisions between gas molecules. These enhanced transport predicted by theoretical studies cannot be verified experimentally due to complications in preparation and characterization of self-supporting CNTs with uniform pore size and distribution [66]. In addition, CNTs have a strong affinity with CO_2_ and it has been reported that they can have a double adsorption capacity compared to activated carbon, even though the increase in surface area is limited to 25% [67].

Exploitation of CNTs in the fabrication of hybrid membranes for gas separation can be achieved by following two different approaches either by aligning the nanotube along with the gas flux and exploiting the high gas diffusion within the inner wall of the nanotube, or by taking advantage of their superior CO_2_ adsorption capacity and reinforcement ability in polymer nanocomposites. In the first case, specific procedures must be followed in order to align the nanotubes properly along with the transmembrane flux. Filtration methods were adopted to align both SWCNTs and MWCNTs by various authors that resulted in enhanced separation performances [61,68,69]. Kim et al. [61] reported the first ever gas mixture transport properties of CNT-membranes. The SWCNTs were aligned vertically in a polysulfone matrix by the shear forces of the solvent flow along with repulsive forces between the carbon nanotubes and the polymer filter surface. The hybrid matrix followed non-Knudsen behavior for CO_2_/CH_4_ mixed gas separation since both species adsorbed on surface of the nanotubes, but CH_4_ adsorption was lowered by the strong CO_2_ competition on the adsorption sites of the nanotubes. However, despite the enhanced selectivity measured for mixed gas conditions, low selective features (α_CO2/CH4_ < 2) were observed for the CNT-based membrane. Zhang et al. [69] aligned the CNTs vertically in the direction of the gas transport in parylene-C matrix to study the mechanism of light gas transport (Table 4). The ends of vertically aligned CNTs were ensured to be open by plasma etching of the surface. The study established that owing to the inherent smoothness of the inside of CNTs the transport was 30 folds faster than the Knudsen model prediction while the selectivity (α_CO2/N2_ ≈ 0.9, α_H2/CO2_ ≈ 4) agreed to Knudsen selectivity for each gas pair contrasting to study by Kim et al. [61]. In most case, the embedment of the CNTs in the polymer matrix is mainly to improve the membrane performance by increasing the CO_2_ solubility in the hybrid matrix, which is related to the CNTs’ surface affinity with CO_2_ and the polymer phase. Despite gas transport through vertically aligned nanotubes has also been demonstrated, their extremely low selective features make them not of particular interest for gas separation applications [69]. For this reason, CNTs have only been listed in the group of particles in fabricating nanocomposite membranes.

When stochastic dispersion of CNT in the hybrid matrix is targeted, the dispersion of CNT in the polymeric solution is obtained by mechanical stirring and/or sonication. Cong et al. [70] studied the effect of addition of SWCNTs and MWCNTs in brominated poly(phenylene oxide) (BPPO). Nanoscale aggregates were found in the membrane casted from the polymer solution containing CNT. The diffusivity of CO_2_ was found to increase with the addition of 5 wt % of SWCNTs, but the variation is claimed to be the result of poor compatibility between the polymer and CNT walls rather than to the enhanced transport within the inner volume of the embedded nanotubes. –COOH functionalized SWCNTs were found to have better compatibility with the polymer phase, reducing the impact on the gas diffusivity, and, despite the limited effect on the gas transport properties, the CNTs increased the mechanical strength of the membrane. An increase in CO_2_, N_2_, O_2_ and CH_4_ diffusivities within 10% to 30% of the pristine polymer value has been obtained by adding 2% CNTs in poly(imide siloxane), due to an increase in free volume [71]. However, at high CNTs loadings (10 wt %) the enhancement is offset by the increased tortuosity around the entangled CNTs domains preventing further increase in permeability and selectivity. In another interesting study [72], the authors revealed the increased CO_2_ uptake capacity of hybrid membranes with embedded CNTs through a theoretical approach, but negligible enhancement was observed experimentally for SWCNTs loading larger than 5 wt %. This was attributed to the fact that at higher loading, the polymer chain packing around the nanofiller interface becomes constrained than free to reorient. On the other side, the CO_2_ diffusivity increased up to 95% (from 1.17 × 10^−8^ cm^2^/s to 2.12 × 10^−8^ cm^2^/s) at 10 wt % SWCNTs loading. Addition of MWCNTs was also found to increase the fractional free volume due to generation of micro voids in the polymer matrix [73,74,75]. Murali et al. [73] reported exceptional increase in separation properties upon addition 2% and 5% MWCNTs in PEBAX-based membranes. The CO_2_ permeability increased from 55.9 to 329.7 Barrer for 2 wt % MWCNTs loading, coupled with a selectivity increase from 40.2 to 78.6. The observed improvement has been mainly attributed to the fractional free volume increase (from 2.6% to 7.2%). Additionally, MWCNTs were also found to decrease the crystallinity in PEBAX membranes, which led to increase in intersegmental spacing and hence more amorphous domains, as confirmed with XRD analysis. Similar reduction in crystallinity and enhanced polymer spacing was observed by Weng et al. [74] upon addition of MWCNTs to poly(bisphenol A-co-4-nitrophthalic anhydride-co-1,3-phenylene diamine) (PBNPI) matrix. Interestingly, the authors reported the increase of CO_2_ permeability of the hybrid membrane related to increased diffusion coefficient, whereas negligible effect on CO_2_/CH_4_ selectivity was observed. Wang et al. [75] studied influence of MWCNTs on the transport properties of PEBAX-PEG blends. In this case, the CNTs increased both CO_2_ diffusivity and solubility of all blends owing to the combined effect of polymer chain disruption, thereby enhancing the free volume and decreased crystallinity of PEBAX matrix. With about 5 wt % loading of MWCNTs on blend of PEBAX with 40 wt % PEGDME, the hybrid membranes overcame the Robeson upper bound, reaching a CO_2_ permeability of 743 Barrer and CO_2_/N_2_ selectivity of 108 (pressure = 10 bar, T ≈ 25 °C). The study also established that high molecular weight PEG in the blends increased the CO_2_/CH_4_ selectivity, while low molecular weight PEG enhanced the CO_2_/N_2_ selectivity. Thus, the increase in transport properties was also attributed to the increased diffusivity and solubility that arise from combined effect of using a secondary component like PEG when CNTs are added in glassy polymers [75].

In general, functionalization of the CNT surface could result in prevention of aggregation and better compatibility of CNTs with the polymer matrix. An interesting study [76] about the functionalization with various metals of the inner or outer wall of CNTs revealed that the gas diffusion pathways are mainly located outside the CNTs, as the CO_2_ permeability was not affected when metal-based nanoparticles were incorporated in the interior side of the nanotube. On the other hand, functionalization of the external surface played a significant role in adsorption of gases affecting the solubility and diffusivity of the transport species. Wong et al. [77] grafted poly(methyl methacrylate) (PMMA) on MWCNTs prior to incorporation in a selective layer fabricated through interfacial polymerization. The hybrid thin film composite membranes increased the CO_2_ permeance of the neat polymer by 29% to 70.5 GPU while simultaneously increasing CO_2_/N_2_ and CO_2_/CH_4_ selectivity by 47% and 9% with respect to neat polymer to 67 and 29, respectively. The enhancement is attributed to increasing CO_2_ affinity towards amine groups and amide groups in the hybrid matrix.

For facilitated transport membranes, the addition of CNTs was mainly oriented towards achieving better mechanical properties. Nevertheless, the CNTs were found to play a dual role by enhancing the strength of the matrix and increasing the water uptake due to the micro voids formation or the filler spacing effect. Deng and Hägg [78] reported the use of CNTs in PVAm/PVA matrix for high pressure CO_2_/CH_4_ separation to obtain enhanced swelling and superior transport properties. The permeance of the membrane increased from 110 GPU to 130 GPU while simultaneously increasing the CO_2_/CH_4_ selectivity from 25 to 45. CNTs have been also employed in order to exploit the enhanced swelling properties in membrane containing CO_2_-philic enzymes [79]. Zhao et al. [80] and Ansaloni et al. [81] introduced MWCNTs and amine functionalized MWCNTs in facilitated transport membranes in order to improve the mechanical stability of the selective layer under high-pressure and high-temperature conditions. The amino-functionalization of the CNTs improved the compatibility with the hydrophilic polymer phase, preventing the performance drop at high filler loading (up to 7 wt %). Stable performance for more than 250 h were reported at a feed pressure of 15 bar.

Table 4 shows the summary of CNT-based nanocomposite membranes presented here above. From the table it can be observed that the loading of CNTs was limited to 5 wt % in most of the studies, mainly due to the increase in tortuosity of gas diffusive pathways, which negatively affects the gas permeability. Small amounts (<5 wt %) of CNTs can contribute to the enhancement of the separation performances in a nanocomposite membrane due to the interaction with the polymer chains leading to a reduced packing density and the formation of nanocavities at the polymer/nanotube interface.

### 3.2. Polymeric Nanofibers

**Figure 7:**
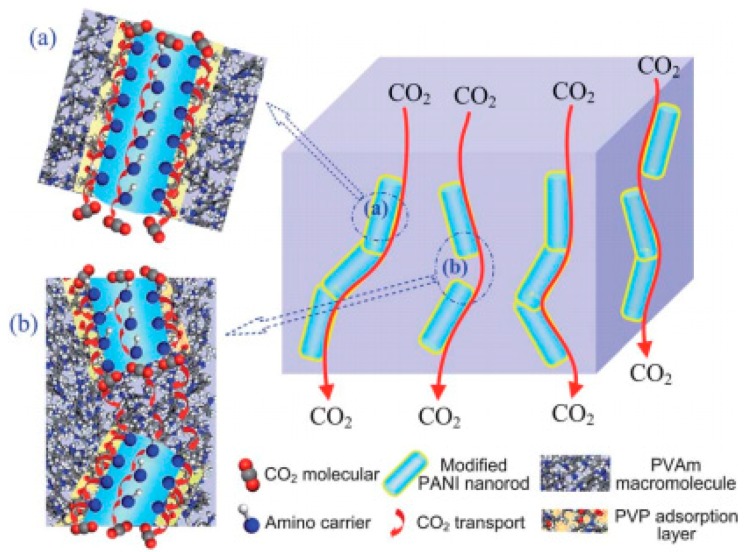
Intrachannel (**a**) and interchannel (**b**) CO_2_ transport in PANI-PVAm/PSU nanocomposite membrane [82].

Nanorods (or nanowire) obtained from poly aniline (PANI) can also be considered part of the 1D family of nanomaterials used for the fabrication of hybrid membranes for gas separation. The presence of amine groups in the molecular chain confers an extremely high CO_2_ adsorption capacity, and the ability to fabricate nano-size 1D particles makes them particularly effective nanofillers for CO_2_ capture applications. Zhao et al. [82] successfully incorporated polyaniline nanorods modified with PVAm polymer chains into PVAm matrix and coated the nanocomposite over a PSU support. A drastic improvement of gas permeability and selectivity was observed. In particular, under humid conditions the CO_2_ permeance increased by 14.4 times to 3080 GPU at 1.1 bar with a CO_2_/N_2_ selectivity increasing by 3.5 times to 240 when compared to neat PVAm matrix for a 2 wt % loading. The work proposed the existence of fast transport channels formed by PVAm containing PANI by means of interchannel and intrachannel CO_2_ transport as shown in Figure 7. In another study [83], Zhao and his co-workers compared the effect of PANI nanorods and nanosheets on the hybrid membranes. The pure gas results of hybrid membrane containing 17 wt % PANI nanofibers in PVAm coated on PSU yielded a CO_2_ permeance of 986 GPU and CO_2_/N_2_ selectivity of 83.4, while the pristine PVAm/PSU had a CO_2_ permeance of 135 GPU and CO_2_/N_2_ selectivity of 51.8. In the case of PANI nanosheets further increase to a CO_2_ permeance of 1400 GPU and CO_2_/N_2_ selectivity of 219.3 at 17 wt % loading has been documented. This enhancement in transport properties has been attributed to the combined effect of increased free volume in the hybrid matrix and intrinsic high CO_2_/N_2_ selectivity of PANI nanoparticles. 

The use of nanocellulose fibrils as engineering material in a wide range of products is being increasingly explored. The motivation behind such interest is attributed to the bio-based nature of the polymer, which has the potential of reducing environmental impacts and carbon footprint in its production process. Additionally, the presence of immense number of hydroxyl groups on the surface of nanocellulose materials paves ways for further functionalization while simultaneously contributing to the hydrophilicity of the biopolymer. For some years, the use of nanocellulose in gas separation applications has been confined to barrier materials due to their inherent resistance to permeation of gases. However, a study by Ansaloni et al. [84] revealed that despite the low permeability of CO_2_, nanocellulose films showcased extremely high CO_2_/N_2_ and CO_2_/CH_4_ selectivity (in the order of 500 and 350 respectively) under humid conditions. The study also explored addition of nanocellulose to hydrophilic Lupamin (PVAm) matrix to enhance the CO_2_ permeation performance. The corresponding hybrid matrices were obtained by mixing the nanocellulose fibers in polymer solutions using a three-roll mill. Venturi et al. [85,86] studied the water vapor solubility of hybrid nanocellulose PVAm membranes at different relative humidity conditions and correlated gas separation performances using simulated results obtained from Park Model. The hybrid membranes showed highly selective matrices for both CO_2_/N_2_ and CO_2_/CH_4_ separations (about 100 for CO_2_/N_2_ and 22 for CO_2_/CH_4_ respectively) transcending the upper bound with a permeability of 130 Barrer at 30 wt % loading of nanocellulose fibers at humid conditions. Results obtained from nanofibers-based hybrid membranes are reported in Table 4. In an attempt to increase the CO_2_ permeance of nanocellulose films, Matsumoto and Kitaoka [87] added nanoporous ZIF-90 MOFs on TEMPO-oxidised nanocellulose fibers to cast hybrid films of nanocellulose. The MOF crystals were found to be closely entangled with the nanofibers where initially the Zn ions associated with the carboxyl groups and later became crystal growth nuclei. These hybrid films significantly decreased the permeance of non-interacting CH_4_ while increasing the CO_2_ permeance from 36.8 GPU to 2970 GPU with a ZIF-90 loading of 44.2 wt % while exhibiting a maximum CO_2_/CH_4_ selectivity of 123 for a 50 µm film. This enhancement is due to generation of large number of nanoholes in the nanocellulose matrix associated to the use of MOFs nanoparticles. 

## 4. Two-Dimensional (2D) Nanofillers

Recent advances revealed several interesting class of 2D fillers in composite materials, including ultra-thin graphene, graphene oxide and MoS_2_, due to their ultra-low thickness and their outstanding mechanical and chemical stability [88]. The high aspect ratio of these 2D materials is suggested to be more suitable for the achievement of higher separation performance compared to isotropically shaped (0D) and 1D materials, as lower loading can have a much larger influence on the transport properties [88]. In addition, with the latest developments in the field of graphene-based materials, tailoring defects on individual graphene monolayers and the possibility of functionalization of the 2D nanosheets have had a positive impact on the usage of graphene as nanofillers in nanocomposite membranes [89,90,91,92,93]. Miculescu et al. [93] recently reviewed the graphene-based nanocomposite membranes with the focus on polysulfone and cellulose based membranes and stated that the application of graphene-based materials is still in its early stage and it is expected to exponentially grow in the upcoming years.

### 4.1. Graphene and Derivates

Monolayer graphene was first discovered in 2004 [94] and immediately its great potential in various fields has been clear, due to its outstanding specific area, Young’s modulus and other physical and chemical properties. In view of the molecular sieving capabilities of nanoporous graphene, ultra-thin graphene membranes for CO_2_ separation is still being investigated by tailoring pore structure in defect-free selective layers [95]. Even though defect-free graphene platelets are impermeable to gases, the use of some production techniques (such as chemical vapor deposition) or of stuck graphene layers can introduce sieving effect among the features of graphene-based nanofillers. Pristine graphene has been used in the preparation of nanocomposite membrane for gas separation, showing beneficial effect on the gas permeability at extremely low loading and on slowing aging phenomena. Furthermore, graphene-based nanomaterials like graphene oxide (GO), reduced graphene oxide (rGO), porous GO (PGO) and functionalized GO have been increasingly studied for incorporation in polymeric membranes for gas separation.

**Figure 8:**
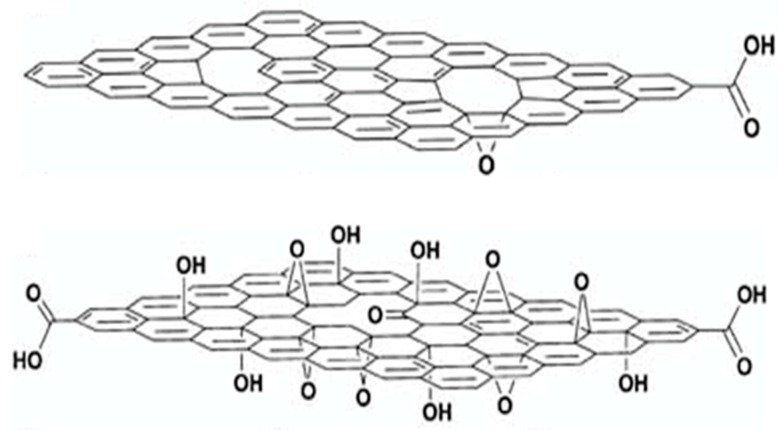
Graphene and graphene oxide nanosheets [96].

Among the graphene-based materials, GO nanosheets have been mostly used as nanofillers in fabricating nanocomposite membrane for enhanced CO_2_ separations, as the abundant number of polar groups (–OH, –COC–, and –COOH) on the basal plane and corners of the sp^2^-hybridised carbon nanosheets benefits CO_2_ transport, as seen in Figure 8. The presence of the polar groups also makes the fillers more compatible with polymer matrices due to the increased interaction sites. When embedded within polymer matrices, the selective interactions of these sites towards CO_2_ enhance solubility-selectivity of the hybrid membranes. Owing to the CO_2_-philic nature, GO nanoplatelets showed extremely high CO_2_-sorption capacity (about 1.5 mmol/g at 1 bar and 273 K), comparable with the one reported for MOFs (0.5 to 1.9 mmol/g) [97].

In the past years, the inherent affinity property of GO nanosheets towards CO_2_ has been exploited to fabricate membranes with superior permeation performance. Inorganic membranes made of few layered GO nanosheets with tunable properties have been studied and found to have outstanding potential in gas separation [98]. Furthermore, GO nanosheets have been conveniently tuned prior to incorporation in polymer matrices to form hybrid membranes. With developments in the chemistry of GO nanosheets, functionalization with various CO_2_-philic moieties has also been attempted. Recently, GO nanosheets have been used as “nano-substrates” to grow nanoparticles like MOFs, preventing their aggregation in the polymer matrix. These tailor made-nanofillers combining GO and MOFs have been found to further enhance CO_2_ separation properties in hybrid membranes.

#### 4.1.1. Pristine Graphene Nanosheets 

Few research studies are present in literature about pristine graphene nanosheets, and focus mainly on high free volume polymers, such as PTMSP [99] and PIM-1 [100]. In most cases, the graphene nanoflakes were dispersed in polymer solutions by sonication or physical mixing. Olivieri et al. [99] embedded a similar amount of industrial grade graphene (XT-IND-G, 0.2 µm × 2–20 nm), research grade graphene (XT-M60-G, 5 µm × 2–8 nm) and graphene oxide (GO, 2 µm × 1.1 nm) in PTMSP and characterized the transport properties of the hybrid membranes. For both graphene grades, a decrease of CO_2_ permeability is observed with negligible effect on gas selectivity (Table 5). In the GO case, a 7% increase of CO_2_ permeability was observed, due to the simultaneous enhancement of CO_2_ solubility and diffusivity in the hybrid matrix. Interestingly, XT-M60-G and GO were also found to affect the free volume relaxation over time, slowing down aging phenomena of the hybrid matrix compared to the case of pristine PTMSP. In-house prepared few layers graphene (FL-G) was also dispersed within PIM-1 (concentration up to 0.024 wt %) and the gas transport properties of the hybrid membranes were thoroughly investigated [100,101]. Interestingly, an optimum of CO_2_ permeability (2.5-fold enhancement compared to the pristine PIM-1) was observed at extremely low loading (~0.001 wt %), suggesting that low amounts of graphene can be beneficial for the transport properties. Nevertheless, the positive effect on permeability was balanced by a decrease of the selective feature of the hybrid matrix (~20% for CO_2_/N_2_ and ~40% for CO_2_/CH_4_). Similar to the case of PTMSP, graphene was shown to be able to reduce the effect of aging on the separation performance in PIM-1. Finally, Rea et al. [102] reported the effect of embedding commercial grades of graphene (XT6, 5 µm × 6–8 nm; XT7, 20 µm × 2 nm) on poly(2,6-dimethyl-1,4-phenylene oxide) (PPO). Surprisingly they were able to reach a loading as high as 15 wt %, but the increasing amount of filler resulted in a drop in the gas permeability values, due to the increased tortuosity of the gas diffusive pathways in the matrix with embedded nanofillers. The results clearly indicate that small amounts of graphene are preferable to improve the separation performance of hybrid membrane.

#### 4.1.2. GO Nanosheets

Shen et al. [103] illustrated the use of GO platelets for fast and selective transport of CO_2_ resulted from the pronounced hydrogen bonding between the nanosheets and PEBAX (crosslinked polyether block amide) polymer. The oxygen atoms present in the highly polarized groups serve as negative center to generate hydrogen bonding with the protons of polymer chains. The interactions further increased with the degree of oxidation (O/C ratio) in the GO nanoplates. The resulted hybrid matrix showed enhanced CO_2_ permeation of up to 100 Barrer and CO_2_/N_2_ selectivity of 91 with significant retention of its properties for long-term operations for about 100 h. The increase in transport performance has been partially attributed to the enhanced CO_2_ solubility, which was about 2.2 times higher compared to the pristine polymer. Thin composite of GO-based hybrid membrane have been developed by dispersing GO in poly(ethylene oxide)–poly(butylene terephthalate) copolymer (known as Polyactive) copolymer [104]. The hybrid selective layer exhibited enhanced selectivity due to the increased tortuosity in the hybrid matrix induced by the embedded inorganic phase. However, the gas permeability decreased with loading, showing an optimum value in terms of separation performance at a GO loading of 0.05 wt %, which transcended the Robeson upper bound for CO_2_/N_2_ separation. Interestingly, it has been reported that an increase in lateral size of the dispersed GO nanoplates embedded within PEBAX^®^ 1657 (crosslinked polyether block amide) was found to affect the stacking of polymer chains compared to the pristine matrix [105]. GO nanosheets with relatively large lateral sizes (5–10 µm) were found to affect negatively the gas transport, as the large size could possibly force alignment of nanofiller perpendicular to direction of penetrant transport pathway. On the other hand, nanosheets with small lateral dimensions (100–200 nm) were found to have less effect on the CO_2_ permeability and CO_2_/N_2_ selectivity due to the inability to steer adequate polymer chain realignment. Optimal design resulted in GO nanosheets with 1–2 µm lateral dimension that yielded superior and stable gas permeation performance, increasing the permeability from about 48 to 92 Barrer with increase in CO_2_/N_2_ from about 38 to 84. The mixed gas performance of the same hybrid materials resulted in further improved permeance and selectivity at 0.1 wt % loading of GO when operated under humid conditions, which remained stable over 100 operating hours. Another study by Zhao et al. [106] revealed that good dispersion of GO nanosheets in PEBAX (crosslinked polyether block amide) matrix could result in increased crystallinity and elasticity modulus of hybrids with increasing loading, while the CO_2_ permeability decreased with respect to the neat polymer. Additionally, Lape model [107] was used to conveniently predict the gas separation performance of GO-loaded hybrid matrices which accounts for the influence of the geometrical shape of the filler on the permeation performance. The model has been widely used for prediction of drop in gas permeability of barrier films, upon addition of impermeable flakes with a specific size distribution. Quan et al. [92] reported the use of high aspect ratio GO nanosheets in cross-liked PEO matrix. Thanks to the good interface compatibility, the study demonstrated tremendous increase in tensile strength and young’s modulus of the hybrid membranes when compared to pristine polymer membranes. Furthermore, the addition of GO was found to increase the fractional free volume due to a reduced interchain entanglement density: the fractional free volume (FFV) increased from 4.291% to 4.773% when 1 wt % GO was added to the pristine matrix. Consequently, the gas permeability improved from 250 to 450 Barrer, together with an increase of CO_2_/N_2_ selectivity from 48 to 55 (Table 5). However, increasing GO loading to over 1 wt % led to a significant drop in diffusivity, thus a decrease in the permeability of the penetrants.

#### 4.1.3. Functionalized GO Nanosheets

Although GO increases solubility of CO_2_ in the hybrid matrix by eliciting spatial realignment of polymeric chains around the nanosheets, the increase in tortuosity due to the intrinsic barrier effect of GO often results in a reduction of permeability. To compensate this loss in gas diffusivity, researchers have dedicated their attention to increase the penetrant solubility by enhancing the CO_2_ adsorption onto the GO surface by functional modification with CO_2_-philic groups [90]. Functionalization of GO also helped in obtaining better dispersion of nanofillers in polymer solutions.

Li et al. [108] dispersed GO nanosheets functionalized with blend of PEG and PEI in PEBAX (crosslinked polyether block amide), reporting an increase in diffusivity selectivity and solubility selectivity due to the presence of amine moieties. The maximum performance was shown at 10 wt % loading, where a CO_2_ permeability of 145 Barrer and selectivity of 62 and 24 for CO_2_/N_2_ and CO_2_/CH_4_, respectively, have been achieved. Humidified permeation tests resulted in further improvement of the performance, owing to the facilitated transport mechanism offered by the amine carriers present in PEI-functionalized GO. CO_2_ permeability (1330 Barrer, +270%) and CO_2_/N_2_ selectivity (120, +240%) leading to promising separation performance (Table 5). Imidazole functionalized GO has been reported by Dai et al. [109] in PEBAX (crosslinked polyether block amide) membranes. Despite the limited effect on CO_2_ permeability (Table 5), a tremendous improvement of CO_2_/N_2_ selectivity (from 65 to 91) was observed at 0.8 wt % loading, while increasing the mechanical properties simultaneously. The modification of GO nanoplates with Zn ions have also been reported to produce hybrid membrane using PEBAX as polymer phase [110]. Dopamine (DA) was used as initiator of the Zn linkage on the GO substrate surface by complexing with the catechol functional groups of DA with the metal ions. In addition to the improved separation performance at low pressure (Table 5), the hybrid membranes also exhibited superior performance for high-pressure CO_2_/CH_4_ separation, with long term operational stability of over 100 h.

Xin et al. [111] reported the functionalization of GO with amino acids compounds (first grafted with dopamine and then cysteine), incorporated in hydrophilic sPEEK. Both primary amines and carboxylic amine groups have been found to increase the CO_2_ solubility under humid conditions, owing to a selective CO_2_-reactivity. The nanofiller addition contributed in achieving superior mechanical and thermal stability compared to the neat polymer. The humid gas permeability showed a 2-folds enhancement (from 565 Barrer to 1247 Barrer), associated with the simultaneous increase of CO_2_/N_2_ and CO_2_/CH_4_ selectivity at 8 wt % loading (Table 5). This increase was attributed to a comprehensive effect of increased water uptake, facilitated transport by presence of amine groups, increased free volume and decreased crystallinity. Another use of functionalized GO in facilitated transport membranes was documented by Shen et al. [112] with hyperbranched PEI in PVAm matrix. The hybrid matrix exhibited a maximum CO_2_ permeance of about 36 GPU at 2 wt % loading of the functionalized filler and a maximum CO_2_/N_2_ selectivity of 107 at 3 wt % loading. The study resulted in superior permeation performance where GO also contributed to long-term stability of the membranes over 100 h. The authors were the first to propose that the interlayer spacing between neighbouring nanosheets could be tailored by incorporating nanoparticles such as ZIFs or nanotubes. 

In general, from the analysis of the reported results it appears clear that by using functionalized GO nanosheets the optimum membrane composition is obtained at a higher nanofillers loading. This is related to the fact that the functionalization acts towards improving the interactions between the CO_2_ and the nanoparticles, therefore higher filler concentration in the hybrid matrix is achievable without significant loss in permeation performance.

#### 4.1.4. Porous GO Nanosheets

Porous graphene oxide has been reported as an easy approach to reduce the gas diffusional resistance of the nanoflakes. The dispersion of tuned porous GO in a PEBAX (crosslinked polyether block amide) matrix [113] determined a 2-fold enhancement of the CO_2_ permeability (from 60 to 119 Barrer) and CO_2_/N_2_ selectivity from 55 to 104 at relatively high GO loading (5 wt %). Zahri et al. [114] demonstrated the first GO-based hollow fiber hybrid membranes with PSU as the selective layer. The study showed an increase in T_g_ with loading of GO, thereby reducing the risks of plasticization and a slight increase in tensile strength and elongation at break. The permeance of the hybrid hollow fiber membranes (0.25 wt % loading) increased from 65.2 to 74.5 GPU at 5 bar and 25 °C while increasing the CO_2_/N_2_ and CO_2_/CH_4_ to about 44.4 and 29.9. To the best of the authors’ knowledge, the pore architecture in the porous GO has not been reported to selectively sieve gaseous penetrants based on their kinetic size. Porous GO in the hybrid membrane still functions as nanofillers, not as an additional matrix. Therefore, the hybrid membranes containing porous GO are grouped under nanocomposite membranes.

#### 4.1.5. Graphene Oxide as Scaffolds for Other Nanoparticles

Another promising approach to optimize the GO functionalization is the bonding of CO_2_-adsorbents on the nanosheet surface. Dong et al. [115] fabricated ZIF-8/GO nanosheets, growing ZIF-8 nanoparticles on reactive sites of GO, and dispersed them in PEBAX (crosslinked polyether block amide) matrix. Owing to high CO_2_ adsorption on ZIF-8, the resulting hybrid matrix showed improved gas transport compared to the pristine sample, with CO_2_ permeability and CO_2_/N_2_ selectivity increasing of 190% and 175%, respectively. The inherent ultramicroporosity of ZIF-8 is claimed to have increased the solubility selectivity of the hybrid matrix while the high aspect ratio of GO contributed to increase in diffusivity selectivity. Lee et al. [116] developed high surface area ZIF-8 on porous GO by hydrothermal treatment of GO to generate mesopores, followed by growth of nanoparticles. Incorporation of these high surface area nanofillers in PEBAX matrix coupled with the affinity to ZIF framework towards CO_2_ resulted in an increased diffusion coefficient at a very low loading (0.02 wt %), leading to an increase in CO_2_ permeability from 83 to 183 Barrer with no loss in the CO_2_/N_2_ selectivity. Furthermore, the porous GO sheets decreased effect of plasticization with no apparent drop in selectivity of the hybrid matrix up to 5 bar transmembrane pressure.

**Figure 9:**
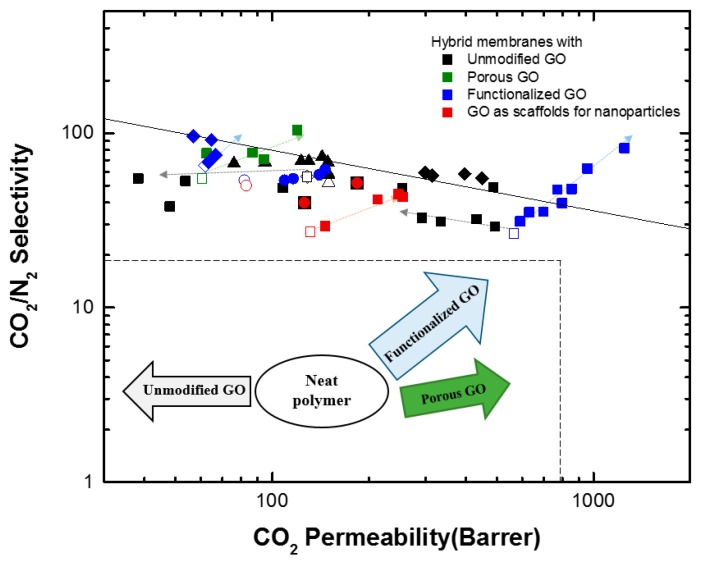
Performance of graphene-containing nanocomposite membranes for CO_2_/N_2_ separation with respect to the upper bound from Robeson 2008 [7]. Empty shapes represent the performance of neat polymer matrix.

The effects of GO nanosheets in the nanocomposite membranes are summarized in the Robeson plot, as shown in Figure 9. Structural modification like porous GO sheets has reduced the diffusional resistance and increased permeability with little change in selectivity. On the other hand, functionalization with CO_2_-philic moieties or using GO-nanosheets as scaffolds for CO_2_-philic nanoparticles has led to simultaneous increase in permeability and selectivity.

### 4.2. Molybdenum Disulfide (MoS_2_)

**Figure 10:**
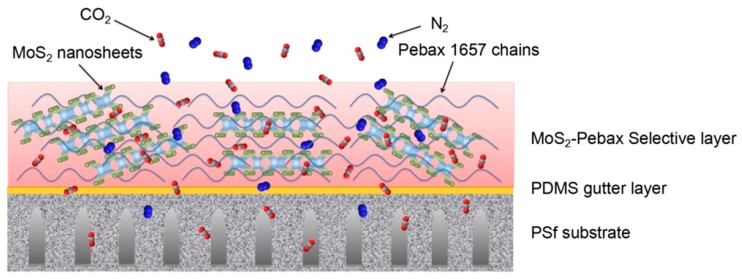
Mechanism of gas transport through MoS_2_ containing hybrid membrane [119].

Another new type of 2D material, MoS_2_, has been recently developed and used as nanofiller in membranes. These 2D transition metal dichalcogenides are metal atoms in a layer sandwiched between sulphur atoms with perfect crystallinity, which are found to have high affinity towards CO_2_ [117]. Since MoS_2_-based hybrid membranes are relatively new, there exist only a few studies in gas separation applications [118]. Shen et al. [119] fabricated a novel thin film composite membrane with MoS_2_ incorporated PEBAX (crosslinked polyether block amide) selective layer coated over a PDMS gutter layer supported on PSU, as seen in Figure 10 through drop coating method. The exfoliation of the MoS_2_ nanosheets was achieved by sonication to result in a stable dispersion. The permeance of the hybrid composite membranes decreased with increasing loading. However, due to the strong adsorption energy of CO_2_ on MoS_2_, both selectivity and permeability increased with increasing loading of the fillers (thickness of the selective layer increased with the increasing loading). At 0.15 wt % loading of MoS_2_ nanosheets the permeability of CO_2_ increased from 45 to 65 Barrer with simultaneous increase in CO_2_/N_2_ selectivity from 45 to 93. The addition of the nanosheets also contributed to the stability of CO_2_ permeance over a period of operation about 50 h.

## 5. Conclusions and Perspective

Nanocomposite membranes for CO_2_ separation has received increased attention due to the strong need for high performance polymeric membranes to overcome the upper bound of polymeric membranes and thus make membrane separation technically and economically feasible for carbon capture. The great innovation in nanotechnology and emerging of novel and advanced materials have accelerated the development of this technology. Over the last two decades, several new nanofillers like CNTs, MOFs, cellulose nanofibers, graphene and its derivatives, MoS_2_ and POSS have been developed and studied increasingly for fabricating hybrid membranes.

Most 0D nanofillers upon addition to polymeric matrices seem to culminate in an increase in CO_2_ permeability. This is mainly due to increased CO_2_ sorption (depending on the filler nature or surface modification) or increased CO_2_ diffusion (due to voids formation at the polymer/nanoparticles interface). In most cases, the effect on CO_2_/N_2_ or CO_2_/CH_4_ selectivity is neutral or negative with respect to increasing loading of the fillers. Several studies have been dedicated to silica-based 0D nanofillers by deeply investigating their effect for CO_2_ separation. Lately, the focus has shifted to POSS nanoparticles, since their large surface area and tunable chemistry allow the incorporation of active functional groups to significantly improve the membrane separation performance. However, homogeneous dispersion of these nanoparticles in a thin selective polymer layer remains the main challenge for the full exploitation of their potentials. Conversely, in view of the good dispersability in polymers and the intrinsic affinity towards CO_2_, metal oxide nanoparticles have been used to prepare nanocomposite membranes with a large variety of polymeric materials. Massive investigation has been dedicated to TiO_2_, due to its CO_2_-philic nature. Other metal oxides with higher CO_2_ adsorption capacity (e.g., ZnO, Al_2_O_3_, MgO) have not yet received the same attention, but have a potential for further development.

Within 1D nanomaterials, CNTs are widely applied to improve the performance of gas separation membranes. The 1D morphology naturally gives a high surface to volume ratio, which gives rise to various possible adsorption/functionalization sites with high binding energy. In general, 1D nanofillers are found to disrupt the polymer chain packing better than the 0D fillers leading to increased permeability of CO_2_, with varying effects on selectivity. Unfortunately, in the case of CNTs, the high mass transport rate within their inner walls could not be leveraged inside polymer matrices, due to complications in alignment and possible entry and end effects. Besides CNTs, promising types of 1D nanomaterials such as polyaniline and cellulose nanofibers have also been investigated for gas separation applications. In the case of PANI nanofibers, at low additive content (e.g., <5 wt %) the gas transport can be improved because of (a) the interaction with the polymer chains leading to a reduced packing density and the formation of nanocavities at the polymer/nanotube interface, or (b) fast transport channels formed due to the presence of the nanofibers. However, higher additive contents (e.g., >5 wt %) negatively affect the gas permeability due to the increased tortuosity. On the other side, nanocellulose fibers own a great potential for breakthrough in gas separation membranes. NCF have been reported to own an extremely high CO_2_ selectivity under humid conditions, but the achievement of high permeability remains a challenge. Moreover, the large amount of functionalizable moieties on the fibers makes NCF the perfect platform to link moieties to improve the CO_2_-philicity of the nano-phase. 

2D materials on the other hand show the utmost significance in the development of new nanocomposite membranes for gas separation. The planar structure is indeed the most suitable nanoparticles configuration for the fabrication of thin selective layers, as it allows the full exploitation of their features, even at low filler loadings. Generally, increase in filler loading beyond a certain limit is found to result in reduction of CO_2_ permeability due to the barrier property of the nanoplatelets. On the other hand, the CO_2_ selective features are found to increase along with the filler concentration. The use of graphene-based 2D materials is mainly related to GO, owing to its stability and the inherent ability to be functionalized. Studies have shown that the addition of a small loading of unmodified GO contributes to changes in polymer chain packing, leading to an increased permeability. In addition, surface modification of graphene derivates has been reported as a successful approach to improve the CO_2_-philicty of the nanoparticles and their compatibility with the polymeric matrix. However, challenges still exist in the up-scaling of the defect free ultra-thin selective nanocomposite membrane coating and other fabrication problems related to the compatibility issues of the embedded material with the polymer matrix.

To achieve the separation performance of nanocomposite membranes needed for different CO_2_ separation applications, future directions should be focused on the following aspects:

- development of new additives with high CO_2_ adsorption capacity and low bending energy, good dispersion property and low cost;
- functionalization of the existing nanofillers to increase the solubility of the active penetrants in the hybrid matrix, and to maximize the compatibility between the two phases in the membrane for a defect-free coating;
- synthesis of new 2D fillers with precise size sieving effect by engineering design the sheets and distance;
- using 2D materials as hosts to disperse MOFs and other nanoparticles with high CO_2_ adsorption capacity to immobilize the nanoparticles that tends to aggregate;
- improving the alignment of the nanofillers to control membrane morphology for better separation performances.

## Figures and Tables

**Table 1:** Classification of nanofillers in hybrid membranes.

Filler Type	Hybrid Membranes with Solid Fillers	
	Nanocomposite Membrane	Mixed Matrix Membrane
0D	Dense Nanoparticles	
	• Silica	
	• Fumed Silica	
	• TiO_2_	
	• MgO	
	• Al_2_O_3_	
	Polyhedral oligomeric silsequioxanes (POSS)	
1D	Carbon nanotubes (CNT)	
	Cellulose nanofibers (CNF)	
	Polyaniline nanorods	
	Zinc nanorods	
2D	Graphene/Graphene Oxide (GO)	
	Molybdenum disulfide (MoS_2_)	
	Polyaniline nanosheets	
3D		Porous nanoparticles
		• Porous silica
		• Porous metal oxides
		Zeolites
		Metal organic frameworks (MOFs)
		Porous organic frameworks (POFs)
		MOF nanosheets *

* While MOF nanosheets are classified as 2D materials based on shape, they are grouped under 3D materials owing to their molecular sieving effect over penetrants.

**Table 2:** Gas separation performance of Silica-based Nanocomposite membranes (operating conditions ranging within 1–3 bar, 20–35 °C, unless differently specified).

Filler	Polymer	Loading (wt %)	P_CO2_ (Barrer)	α_CO2/N2_	α_CO2/CH4_	Ref
	P84 co-polyimide BTDA-TDI/MDI	0	0.9	20.2		[15]
SiO_2_		4	1.2	16.6		
		8	1.3	15.0		
APTES-		4	0.9	19.6		
modified		8	1.1	16.1		
SiO_2_		14	1.3	17.8		
		20	1.6	10.1		
		25	3.0	4.0		
	PEBAX-1657 crosslinked polyether block amide	0	80.2	71.5		[21]
SiO_2_		5	66.0	50.4		
		10	62.9	49.2		
		30	51.4	46.5		
	polyurethane ^a^	0	189.6	25.0	9.7	[22]
silica		2.5	176.2	29.6	10.8	
28 nm		5	160.8	32.3	11.8	
		10	152.2	36.1	12.4	
		20	124.5	39.8	13.1	
	Polycaprolactum/polyurethene ^a^	0	86.3	34.1	15.4	[16]
SiO_2_		2.5	66.8	42.8	18.4	
		5	62.1	45.0	17.5	
		10	59.1	45.5	18.3	
		20	53.6	54.1	18.6	
		30	41.3	62.5	19.1	
	PEBAX-1074 crosslinked polyether block amide	0	110.7		11.1	[17]
SiO_2_		2	116.7		11.1	
		4	121.7		11.1	
		6	134.2		12.0	
		8	152.1		13.3	
	Matrimid BTDA-DAPI polyimide	0	9.9		35.3	[23]
SiO_2_		0.92	21.3		33.2	
200 nm		1.6	41.0		36.6	
		3.11	46.3		28.3	
	PEBAX-2533 crosslinked polyether block amide	0	221.7	26.3	7.9	[26]
		10	252.4	26.2	7.5	
PEG-POSS		20	276.7	27.8	7.9	
		30	297.9	30.0	9.0	
		40	288.9	31.0	8.8	
		50	148.2	34.9	9.5	
	PEBAX-1657 crosslinked polyether block amide	0	74.5	53.0	17.1	
		10	70.7	48.2	17.1	
		20	99.3	50.5	16.2	
		30	150.7	50.9	15.9	
	PIM 1	0	2795	19	12	[27]
PEG-POSS		1	3360	18	13	
		2	3381	22	16	
		5	1875	26	21	
		10	1309	31	30	

^a^ Pressure = 10 bar.

**Table 3:** Gas separation performance of different metal-oxide nanofillers (operating conditions ranging within 1–3.5 bar upstream side pressure, 25–35 °C, unless differently specified).

Filler	Polymer	Loading (wt %)	P_CO2_ (Barrer)	α_CO2/N2_	α_CO2/CH4_	Ref
	PTGMP ^a^	0	18,600	6.7	3.0	[38]
TiO_2_ ~10 nm		5	20,000	6.1	2.4	
		10	24,900	8.0	3.1	
		20	20,400	6.5	2.6	
	PTMSP	0	35,000	3.8	1.59	[32]
TiO_2_ aggregates, 10–50 nm		3 ^b^	27,000	4.6	1.80	
		7 ^b^	30,000	4.8	1.67	
		10 ^b^	33,000	5.9	1.94	
		15 ^b^	35,000	4.9	1.67	
		23 ^b^	56,000	4.3	1.56	
		33 ^b^	71,000	3.6	1.39	
	PMP	0	6700	7.1	3.7	[39]
TiO_2_ 21 nm		15	6980	7.1	3.5	
		25	8430	7.0	3.5	
		35	10,970	6.5	3.2	
	PES	0	2.0		26.0	[40]
TiO_2_ 70 nm		2	2.3		24.8	
		4	2.6		39.6	
		6	2.6		31.1	
		10	2.9		19.8	
		20	5.6		16.8	
	Matrimid BTDA-DAPI polyimide	0	4.3	19.5	20.5	[41]
TiO_2_ aggregates, 3 nm		5	5.4	15.0	16.9	
		10	7.4	14.8	18.1	
		15	8.0	10.8	13.8	
		20	10.5	11.4	13.7	
		25	12.0	8.9	6.5	
	polybutadiene	0	50.8	14.1	6.8	[30]
Brookite aggregates, 2–60 nm		7 ^b^	51.2	16.6	7.1	
		13 ^b^	65.8	17.9	7.7	
		20 ^b^	87.2	15.4	6.6	
		27 ^b^	161.6	15.2	8.4	
	PEBAX-1074 (crosslinked polyether block amide)/PEG	0	150.4		20.8	[42]
TiO_2_ 21 nm		2	154.4		21.3	
		4	159.9		21.8	
		6	179.4		22.6	
		8	204.5		23.6	
	PEBAX-1074 crosslinked polyether block amide	0	110.7		11.1	[17]
TiO_2_ 21 nm		2	111.8		11.1	
		4	117.5		11.2	
		6	125.4		11.7	
		8	150.3		13.2	
	PVA	0	10.5	7.0		[43]
TiO_2_ 21 nm		10	7.4	7.4		
		20	7.0	8.8		
		30	7.0	5.6		
		40	7.3	5.4		
	PVAc	0	2.9	58.7		[44]
TiO_2_ 21 nm		1	4.2	69.4		
		5	4.8	71.8		
		10	5.3	74.4		
		15	5.8	73.9		
	sPEEK ^c^	0	564.0	38.9	28.8	[31]
TiO_2_		5	680.0	39.3	29.8	
		10	835.4	43.1	32.6	
		15	1342.3	34.7	29.1	
Dopamine functionalizedd TiO_2_		5	680.0	42.1	28.3	
		10	1055.1	46.9	29.1	
		15	1342.3	52.7	35.3	
DA and PEI functionalized TiO_2_		5	574.6	44.5	33.2	
		10	1349.7	56.1	39.3	
		15	1632.4	64.4	58.2	
	PEBAX-1074 crosslinked polyether block amide	0	110.7	50.1	11.1	[45]
ZnO		2	120.6	53.1	11.1	
		4	124.7	54.4	11.2	
		6	131.7	57.0	11.8	
		8	152.3	62.2	13.5	
	PEBAX-1657 (crosslinked polyether block amide)/PEG	0	71.7		25.8	[49]
ZnO		4	94.5		23.8	
	PEBAX-1074 crosslinked polyether block amide	0	110.7		11.1	[17]
Al_2_O_3_		2	118.3		11.1	
		4	128.4		11.5	
		6	137.9		12.2	
		8	163.9		14.2	
	PTMSP	0	34,000	6.2	2.3	[52]
MgO		13 ^c^	53,960		1.9	
		30 ^c^	92,477	4.1	1.7	
		40 ^c^	224,358	3.8	1.5	
		50 ^c^	449,604	3.4	1.4	
		75 ^c^	570,425	2.6	0.9	
	PSU	0	7.7	27.7	30.8	[50]
MgO		10	9.4	25.4	27.6	
		20	11.2	24.0	26.7	
		30	14.1	23.6	25.7	
	Matrimid BTDA-DAPI polyimide	0	6.8	25.0	33.3	[51]
MgO		20	7.5	23.0	29.8	
		30	8.5	24.5	26.9	
		40	9.5	19.6	26.4	

^a^ Pressure = 0.2 bar; ^b^ amount of nanoparticles reported in vol %; ^c^ data obtained under humid conditions.

**Table 4:** Gas separation performance of hybrid membranes with 1D nanoparticles (operating conditions ranging within 1–5 bar and 22–35 °C, unless differently specified).

Filler	Polymer	Loading (wt %)	P_CO2_ (Barrer)	α_CO2/N2_	α_CO2/CH4_	Ref
	BPPO ^a^	0	78	30		[70]
SWNT SWNT-COOH		5	123	29		
		5	79	30		
MWNT		5	153	28		
	poly(imide-siloxane)	0	166.0	13.9	5.89	[71]
SWNT		2	190.7	13.2	5.58	
		10	191.3	10.7	5.21	
	PSU	0	3.9	22.4	22.9	[72]
SWNT		5	5.12	22.3	19.0	
		10	5.19	22.6	18.5	
		15	4.52	20.5	16.1	
	PEBAX 1657 crosslinked polyether block amide	0	55.9	40.2		[73]
MWNT		2	329	78.6		
		5	262.2	58.5		
	XL PEBAX 1657	0	13.38	56.9		
MWNT		2	3.54	83.2		
		5	17.47	84.5		
	PBNPI	0	2.6		3.7	[74]
MWNT		2.5	2.7		2.8	
		10	4.9		3.2	
		15	6		3.4	
	PEBAX 1657 crosslinked polyether block amide	0	88.4	49.4	20.4	[75]
MWNT		2	119.3	51.5	17.6	
	PEGDME (90,10)	0	162	56.2	16.5	
MWNT		2	196.7	62	15.4	
	PEG600 (90,10)	0	144.9	48.46	20.5	
MWNT		2	179	52.3	18.9	
	PEG10k (90,10)	0	66.2	44.6	21.3	
MWNT		2	89.6	49.2	17.8	
	PEG20k (90,10)	0	67.7	46.1	21.7	
MWNT		2	102.8	49.9	17.9	
	PEG20k (80,20)	0	48.1	28.2	25.4	
MWNT		2	72.2	32.2	21.0	
	PEG20k (60,40)	0	23.5	18.7	30.1	
MWNT		2	35	22.7	23.7	
	PES	0	2.6	22		[76]
CNT-COOH		5	4.5	22		
CNT-Ru		5	3.6	22.5		
CNT-Fe		10	4.4	11.5		
	PDMS	0	54.9	45.7	26.6	[77]
PMMA-CNT		0.5 ^b^	58.1	34.8	20.6	
		0.5 ^b^	70.5	67.2	29.0	
	PVAm/PVA ^b^	0	110 ^c^		25	[78]
MWNT		1	130 ^c^		45	
	PVA	0	120	60		[79]
MWNT		1	135	60		
	PVA + ME	0	193	103		
MWNT		1	301	120		
	polyamine blends	0	984	283.6		[81]
Amine functionalized	on PSU ^c^	2	943	264.9		
MWNT		6	1013	265.4		
	PVAm on PSU ^d^	0	214	68.6		[82]
PANI nanorods		2	3080	240		
	PVAm on PSU ^d^	0	135.0 ^e^	51.8		[83]
PANI nanofibers		1	203.6 ^e^	82.9		
		8	459.9 ^e^	135.7		
		17	990.6 ^e^	83.4		
PANI nanosheets		1	589.9 ^e^	108.3		
		8	662.1 ^e^	105.2		
		17	1402.2 ^e^	219.3		
NCF	Lupamin ^d^ (PVAm)	30	187	100	88	[85]
		70	62	44	27	
		50	187	48	19	[84]

^a^ pressure = 0.1 bar; ^b^ g/L in organic phase of coating solution; ^c^ data obtained under humid conditions; ^d^ pressure = 15 bar, temperature = 107 °C; ^e^ permeance (GPU).

**Table 5:** Gas separation performance of graphene-based hybrid membranes (operating conditions ranging within, 25–35 °C, 0.5–7 bar upstream side pressure unless differently specified).

Filler	Polymer	Loading (wt %)	P_CO2_ (Barrer)	α_CO2/N2_	α_CO2/CH4_	Ref
	PTMSP		22,400	6.4	2.5	[99]
XT-IND-G		1	16,990	6.7	2.7	
XT-M60-G		1	19,360	6.2	2.3	
GO		1	23,950	6.3	2.4	
	PIM-1	0	5120	19.0	15.1	[100]
FL-G		0.00096	12,700	14.6	8.8	
		0.0018	9840	17.3	12.3	
		0.0034	7830	19.1	14.2	
		0.0071	3410	20.1	21.3	
		0.0243	5150	19.1	13.2	
	PPO		61	20.3		[102]
XT6		1	60	16.7		
		5	51	18.2		
		15	27	15.0		
XT7		0.3	62	17.7		
	PEBAX crosslinked polyether block amide	0	50.5	51.9	21.6	[103]
GO		0.05	59.1	57.8	22.1	
		0.075	72.7	71.7	22.3	
		0.1	100	91	24.6	
		0.4	30			
		0.5	23			
	Polyactive	0	150.0	52.0	18.0	[104]
GO	PEO-PBT	0.025	150.0	58.0	19.0	
		0.05	149.0	68.0	21.0	
		0.065	143.0	73.0	21.0	
		0.075	130.0	69.0	22.0	
		0.125	123.0	69.0	21.0	
		0.25	95.0	68.0	21.0	
		0.5	76.0	67.0	21.0	
	PEBAX 1657 crosslinked polyether block amide	0	48.0	38.0		[105]
GO-S		0.1	52.6	47.6		
GO-M		0.1	92.5	84.3		
GO-L		0.1	14.0	35.3		
	PEBAX 1657 crosslinked polyether block amide	0 ^a^	128.6	56.2		[106]
GO		0.99 ^a^	108.0	48.5		
		1.96 ^a^	53.7	53.1		
		3.85 ^a^	38.3	54.8		
	PEG	0	254.2	48.2		[92]
GO		0.5	397.3	58.2		
		1	449.6	55.0		
		2	314.8	57.1		
		3	299.6	59.3		
	PEBAX 1657 ^c^ crosslinked polyether block amide	0	81.9	53.7	18.7	[108]
PEG-PEI-GO		1	109.3	53.7	21.0	
		3	116.3	54.8	22.1	
		5	140.2	57.8	22.1	
		10	146.0	61.8	24.4	
		12	140.0	57.6	21.1	
	PEBAX1657 ^d^ crosslinked polyether block amide	0	488.4	49.0	15.7	
PEI-GO		10	1086.3	103.3	30.9	
PEG-PEI-GO		10	1334.7	119.6	45.4	
	PEBAX 1657 crosslinked polyether block amide	0	61.9	65.3	26.0	[109]
Imidazole-GO		0.2	63.4	68.4	25.3	
		0.5	66.6	75.0	24.7	
		0.8	64.6	91.3	25.3	
		1	56.8	96.1	29.1	
	PEBAX 1657 crosslinked polyether block amide	0	92.6		17.2	[110]
Zn-DA-GO		0.5	122.1		26.5	
		1	137.8		28.8	
		2	121.0		30.6	
		2.5	116.9		29.2	
	sPEEK ^d^	0	565.3	26.6	38.1	[111]
GO		2	493.2	29.2	39.3	
		4	335.2	31.1	42.8	
		6	292.6	32.7	43.6	
		8	432.7	32.2	41.8	
DA-GO		2	590.5	31.2	46.1	
		4	698.4	35.4	61.5	
		6	798.3	39.7	67.2	
		8	856.2	47.8	74.5	
DA-Cysteine-GO		2	631.1	35.2	48.1	
		4	771.9	47.4	71.5	
		6	957.8	62.7	93.2	
		8	1247.6	81.8	114.5	
	PVAm	0	13.9 ^a^	77.9		[112]
PEI GO		1	8.8 ^a^	82.6		
		2	35.1 ^a^	90.0		
		3	31.0 ^a^	106.9		
		4	27.7 ^a^	80.9		
		5	26.8 ^a^	76.7		
	PEBAX 1657 crosslinked polyether block amide	0	60.5	54.7		[113]
Por-GO		1.67	86.8	77.3		
		3.33	94.1	70.8		
		5	119.5	103.9		
		6.67	62.4	76.8		
	PSU	0	65.2 ^b^	17.3	17.2	[114]
Por-GO		0.25	74.5 ^b^	44.4	29.9	
	PEBAX 2533 crosslinked polyether block amide	0	131.6	27.1		[115]
ZIF-8 on GO		2	146.3	29.4		
		4	212.7	41.7		
		6	247.2	44.7		
		8	254.5	43.1		
	PEBAX 1657 crosslinked polyether block amide	0	83.0	50.0		[116]
ZIF-8 on GO		0.02	125.9	39.9		
ZIF-8 on por-GO		0.02	183.8	51.9		

^a^ amount of nanoparticles reported in vol %; ^b^ Permeance (GPU); ^c^ data obtained under dry conditions; ^d^ data obtained under humid conditions.

## Abbreviations

6-FDA	2,2′-bis(3,4-dicarboxyphenyl) hexafluoropropane dianhydride
6F-PAI	Hexafluorinated poly(amide-imide)
APTES	(3-Aminopropyl)triethoxysilane
BTDA	3,3′,4,4′-benzophenone tetracarboxylic dianhydride
BPPO	Brominated poly(2,6-diphenyl-1,4-phenylene oxide)
CCS	Carbon capture and storage
CNT	Carbon nanotubes
DA	Dopamine
DAPI	Diaminophenylindane
GO	Graphene Oxide
GPU	Gas Permeation Unit
HFBAA	4,4-(hexafluoroisopropylidene) diphenylazide
MDI	MDI
MMM	Mixed Matrix Membranes
MOF	Metal Organic Frameworks
MWCNT	Multi-walled carbon nanotubes
P84	Co-polyimide BTDA-TDI/MDI
PANI	Polyaniline
PBNPI	poly(bisphenol A-co-4-nitrophthalic anhydride-co-1,3-phenylene diamine)
PBT	poly(butylene terephthalate)
PEA	Polyetheramide
PEBAX	Crosslinked polyether block amide (Crosslinked)
PEI	Polyetherimide
PEG	Polyethylene glycol
PES	Polyethersulfone
PIM	Polymers of Intrinsic Microporosity
PMMA	Poly(methyl methacrylate)
PMP	Poly(4-methyl-2-pentyne)
POSS	Polyhedral Oligomeric Silsesquioxane
PSU	Polysulfone
PTGMP	Poly(1-trimethylgermyl-1-propyne)
PTMSP	Poly(1-trimethylsilyl-1-propyne)
PVA	Polyvinyl alcohol
PVAc	Polyvinyl acetate
PVAm	Polyvinyl amine
SPEEK	Sulfonated Poly(Ether Ether Ketone)
SWCNT	Single-walled carbon nanotubes
TDI	Methylphenylene-diamine
TEMPO	[(2,2,6,6-tetramethylpiperidin-1-yl)oxy radical]-oxidation
XRD	X-Ray Diffraction
ZIF	Zeolitic imidazolate framework
